# Identification of whole blood mRNA and microRNA biomarkers of tissue damage and immune function resulting from amphetamine exposure or heat stroke in adult male rats

**DOI:** 10.1371/journal.pone.0210273

**Published:** 2019-02-19

**Authors:** Luísa Camacho, Camila S. Silva, Joseph P. Hanig, Robert P. Schleimer, Nysia I. George, John F. Bowyer

**Affiliations:** 1 Division of Biochemical Toxicology, National Center for Toxicological Research, U.S. Food and Drug Administration, Jefferson, Arkansas, United States of America; 2 Center for Drug Evaluation and Research, U.S. Food and Drug Administration, Silver Spring, Maryland, United States of America; 3 Division of Allergy and Immunology, Northwestern Feinberg School of Medicine, Chicago, Illinois, United States of America; 4 Division of Bioinformatics and Biostatistics, NCTR/U.S. Food and Drug Administration, Jefferson, Arkansas, United States of America; 5 Division of Neurotoxicology, NCTR/U.S. Food and Drug Administration, Jefferson, Arkansas, United States of America; Mayo Clinic Minnesota, UNITED STATES

## Abstract

This work extends the understanding of how toxic exposures to amphetamine (AMPH) adversely affect the immune system and lead to tissue damage. Importantly, it determines which effects of AMPH are and are not due to pronounced hyperthermia. Whole blood messenger RNA (mRNA) and whole blood and serum microRNA (miRNA) transcripts were identified in adult male Sprague-Dawley rats after exposure to toxic AMPH under normothermic conditions, AMPH when it produces pronounced hyperthermia, or environmentally-induced hyperthermia (EIH). mRNA transcripts with large increases in fold-change in treated relative to control rats and very low expression in the control group were a rich source of organ-specific transcripts in blood. When severe hyperthermia was produced by either EIH or AMPH, significant increases in circulating organ-specific transcripts for liver (*Alb*, *Fbg*, *F2*), pancreas (*Spink1*), bronchi/lungs (*F3*, *Cyp4b1*), bone marrow (*Np4*, *RatNP-3b*), and kidney (*Cesl1*, *Slc22a8*) were observed. Liver damage was suggested also by increased *miR-122* levels in the serum. Increases in muscle/heart-enriched transcripts were produced by AMPH even in the absence of hyperthermia. Expression increases in immune-related transcripts, particularly *Cd14* and *Vcan*, indicate that AMPH can activate the innate immune system in the absence of hyperthermia. Most transcripts specific for T-cells decreased 50–70% after AMPH exposure or EIH, with the noted exception of *Ccr5* and *Chst12*. This is probably due to T-cells leaving the circulation and down-regulation of these genes. Transcript changes specific for B-cells or B-lymphoblasts in the AMPH and EIH groups ranged widely from decreasing ≈ 40% (*Cd19*, *Cd180*) to increasing 30 to 100% (*Tk1*, *Ahsa1*) to increasing ≥500% (*Stip1*, *Ackr3*). The marked increases in *Ccr2*, *Ccr5*, *Pld1*, and *Ackr3* produced by either AMPH or EIH observed *in vivo* provide further insight into the initial immune system alterations that result from methamphetamine and AMPH abuse and could modify risk for HIV and other viral infections.

## Introduction

Life-threatening hyperthermia is a serious toxic effect of amphetamines, including amphetamine (AMPH) and methamphetamine (METH), in both humans and laboratory animals [1–5]. In laboratory animals, the magnitude of hyperthermia correlates directly with the severity of neurotoxicity produced by AMPH and METH [6–9]. More recently, amphetamine-related damage to brain vasculature, liver, and kidney, and activation of the innate immune system have been implicated in exacerbating the neurotoxicity produced by amphetamines [10–14]. These effects are likely due, in part, to a hyperthermic response produced by amphetamines. This hyperthermic response is very similar to what occurs in heat stroke [15–18]. However, the effects of amphetamines and stress on neuroinflammation, and how this interaction may alter the neurotoxicity produced by amphetamines are complex and still being evaluated [19–23].

MicroRNAs (miRNAs) are small, 20–24 nucleotide-long endogenous non-coding RNAs that play an important role in the control of gene expression [24–26]. miRNA profiles vary by tissue and can be modulated by the onset and development of toxicities and diseases [27]. They are stable in biofluids, including blood, and the relative concentrations of specific circulating miRNAs are altered upon damage to different organs (e.g. liver, kidney, and heart) or after modulation of the immune system [27, 28]. Thus, several reports indicate the potential of circulating miRNAs as biomarkers of disease or toxicity [29, 30].

We hypothesize that many of the AMPH-produced adverse changes seen in the neuroimmune system [19, 20, 22, 23, 31, 32] will be reflected also in similar immune system changes in circulating blood, such as activation of the innate immune system. In addition, we thought it would be likely that both miRNA and mRNA transcripts would be present in the blood also as a result of AMPH-induced damage to liver, muscle, kidney, and pancreas. Detection of such miRNAs and mRNAs could provide additional biomarker candidates of the toxicity of AMPH and METH. Finally, it was hoped that the results of this study would help to clarify further how activation of immune system components in the circulating blood and damage of the brain vascular due to amphetamine may alter neuroinflammation, neurotoxicity, and infection [33–35].

The present study identifies the initial/early changes in mRNA and miRNA in circulating whole blood to address several issues, including the differences in the neurotoxicity and magnitude of the initial innate immune response between hyperthermic and non-hyperthermic exposures to AMPH, not determined in previous research. Messenger RNA and miRNA profiling was performed using both RNA sequencing (RNA-seq) and quantitative reverse transcription real-time polymerase chain reaction (RT-qPCR). An emphasis has been placed on determining the miRNA and mRNA expression changes, due to high level AMPH exposure, that affect immune function.

Portions of the mRNA RNA-seq data have been used in previous studies for three different research goals: 1) to develop RNA-seq outlier methodology [36]; 2) to evaluate the stability of RNA-seq gene expression [14]; and 3) to evaluate a multi-class classifier for RNA-seq data using a computational evolution system [37]. However, the miRNA expression data and their relevance to AMPH toxicity and the analysis of the mRNA expression changes have not been published before. As well, blood RNA-seq data related to hyperthermia alone (EIH) or a normothermic AMPH exposure have not been previously evaluated with respect to the entire transcriptome profiles and which transcripts are significantly changed by treatment. The present manuscript compares four treatment groups (new analysis) to better understand the range of amphetamine toxicities that can be produced. Because exposure to high doses of AMPH results in hyperthermia, inclusion of a normothermic AMPH group (kept in a 16°C environment to avoid hyperthermia) and an EIH group (which caused severe hyperthermia) enabled the determination of the non-hyperthermic- and hyperthermic-mediated effects of a neurotoxic AMPH exposure, which has not been previously reported. Finally, we have identified organ specific miRNA and mRNA transcripts in blood species that are potential biomarkers of toxicity (not previously reported).

## Materials and methods

### Animal dosing and sacrifice

This study was conducted in accordance with the Guide for the Care and Use of Laboratory Animals as adopted and promulgated by the National Institutes of Health. Animal procurement and testing was done under protocols E7295 and E7519 (issued to John Bowyer) approved by the NCTR institutional animal care and use committee (IACUC), accredited by NIH-OLAW. Nine- to ten-week-old (≈ 65 days) male Sprague-Dawley rats, ninety-five in total, were obtained from the Charles River Laboratories [Crl:CD(SD)], and received unique tail tattoos for identification after arrival at NCTR. Prior to testing, rats were housed two per cage with food and water available ad libitum, and kept on a daily 12 hr light cycle with lights on at 6:00 a.m. and off at 6:00 p.m. The environmental temperature (23°C) and humidity (53%) were controlled. The rats were tested between 12 and 13 weeks of age (≈ 87 days old).

Dosing occurred over a 6 h period starting at ≈ 8:00 a.m. and ending at ≈ 2:00 p.m. AMPH-exposed animals were given a sequential exposure to 5, 7.5, 10, and 10 mg AMPH/kg body weight (bw) subcutaneously with 2 hr between each dose. The AMPH hyperthermic (AMPH hyper) animals were dosed in a 23 to 24°C environment and AMPH normothermic (AMPH normo) animals were exposed in a 16°C environment. The D-amphetamine sulfate (Sigma-Aldrich, St. Louis, MO) was dissolved in normal saline for subcutaneous injection (1 ml/kg bw per injection). This AMPH dosing paradigm was very similar to an acute METH dosing paradigm that is often used to produce neurotoxicity [25, 26]. The saline control animals received 4 subcutaneous injections of 1 ml/kg bw each in a 23°C environment, and the EIH animals received 4 subcutaneous saline injections of 1 ml/kg bw each in a 39°C environment. In all groups, the behavior and body temperature of each rat was monitored hourly and until 3 hr after the last dose. The lethal effects of hyperthermia and hyperpyrexia can occur with AMPH when body temperatures exceed 41.5°C for more than 30 min. This was prevented by placing the animals unrestrained on crushed ice for 15 to 30 min in a clean plastic cage to drop their temperatures to below 40.0°C, as previously reported [38].

**Fig 1:**
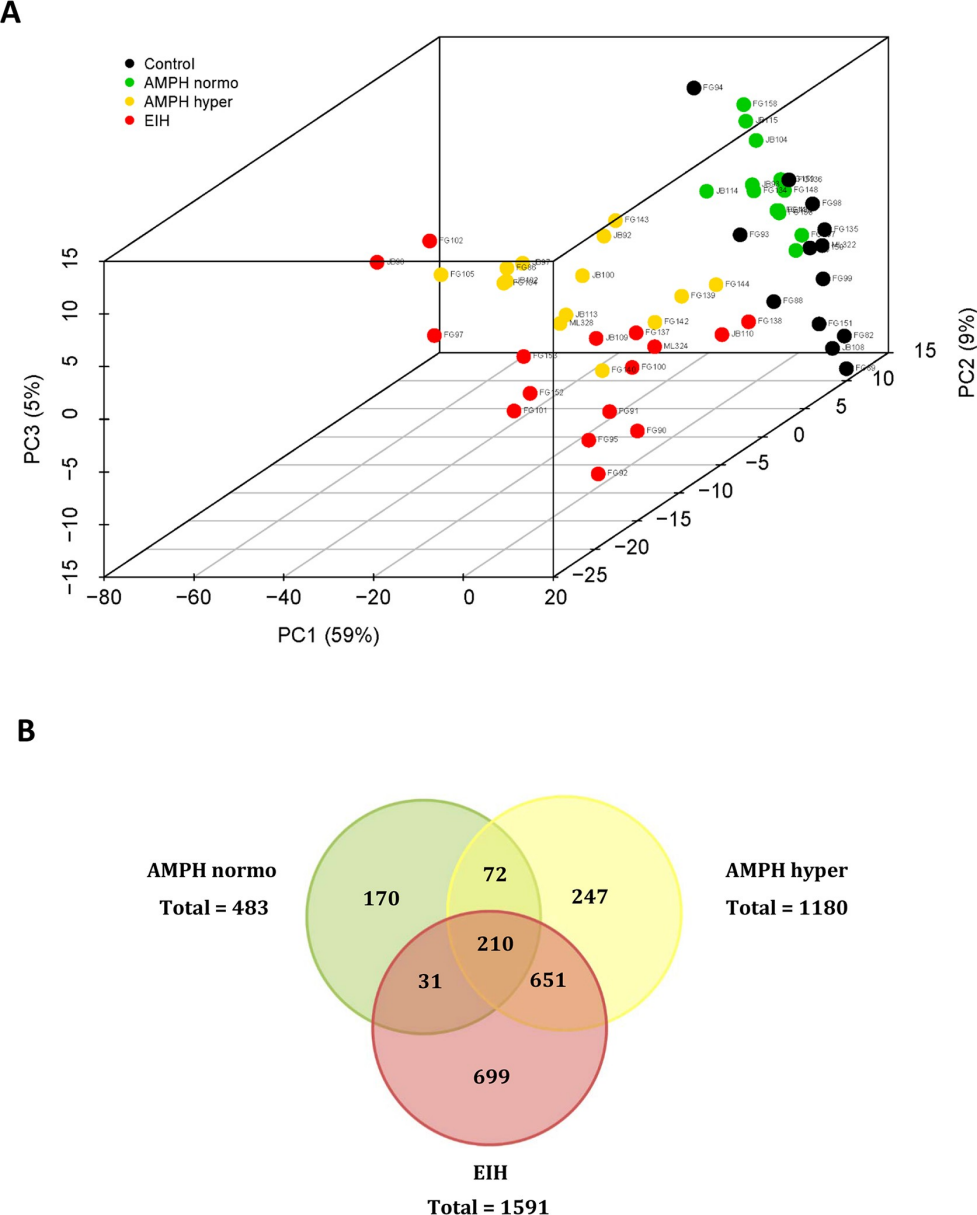
PCA and venn diagram analysis. **A.** Principal component analysis score plot (3 principal components) of AMPH normo, AMPH hyper, EIH, and control treatment groups. **B.** Venn diagram showing the overlap of significant transcripts between the three main pairwise comparisons based on RNA-seq data analysis.

All the data for mRNA expression and miRNA expression determined by RT-qPCR for the four treatments were obtained from three replicates of the same experiment conducted over a 1.5-year period. The multiple replications were to ensure that any significant effects seen in transcript expression would be robust and reproducible over time. A forth additional experiment was conducted, which included only three dosing groups: saline given at 23°C (control), saline given in a cool 16°C environment, and AMPH hyper group. This final experiment was conducted approximately 4 months after the first three replicates. The cool control group was tested due to concerns that there might be a remote possibility of the cool environment affecting mRNA and miRNA profiles relative to the 23°C environment. Blood from the control and AMPH hyper groups were used in a previous publication to help determine the stability of RNAseq transcriptome (Group 4 data in Fig 1) and leukocyte subtype levels [36]. Total RNA from some of the control and AMPH hyper groups in this last experiment were used to generate a sufficient number of animals (n = 7 for both groups) to use RNAseq techniques to quantify blood miRNA levels. This was necessitated to due to depletion of the total RNA in the control and AMPH hyper groups in the first three replicate experiments, due to the RNAseq and RT-qPCR miRNA analysis processes.

### Collection and processing of cardiac blood

Rats were sacrificed 3 hr after the last dose, between 4:00 p.m. and 5:00 p.m., with a lethal dose of approximately 150 to 300 mg/kg i.p. injection of pentobarbital. This resulted in very slow and shallow respiration and no response to pain stimuli, within 3 min. At that point, 3 to 5 ml of cardiac blood was withdrawn from the heart using an 18-gauge needle attached to a 5 ml syringe, and the rats were then euthanatized by decapitation. Approximately 1 ml of the blood was immediately separated into three 300–400 μl aliquots, frozen on dry ice in cylindrical foil capsules, and stored at -70°C for later mRNA and miRNA profile analyses. One ml of the blood was placed on ice (1 to 2 hr) and serum was separated by centrifugation and stored at -70°C until analysis. For many of the controls and AMPH hyper animals, 1 ml of blood was placed in a 2 ml centrifuge tube with EDTA (10 mM final concentration) to determine blood cell type numbers, as previously described [14]. Data on the blood cell counts have been previously reported [14]. Any remaining blood was prepared as previously described for RNA isolation.

### RNA isolation for mRNA and miRNA analyses

Total RNA isolation was performed using RNAzol BD (Molecular Research Center, Inc., www.mrcgene.com) [9] with modified procedures for frozen blood, as previously described [14]. Approximately 300 to 400 μl of frozen whole blood was used for RNA isolation. The final total cellular RNA recovered ranged from 5 to 25 μg and was stored at -70°C. Two to four μl of each aliquot were set aside for purity analysis using an Agilent 2200 TapeStation System (Agilent Technologies, Palo Alto, CA) and the quantity and purity of the RNA were assessed using a Nanodrop ND-8000 instrument (Thermo Scientific). RNA was isolated from 50 μl of serum using a MagMAx mirVana total RNA isolation kit and a King Fisher Flex purification system (Thermo Scientific).

A frozen aliquot of whole blood total RNA was shipped overnight on dry ice to Expression Analysis Inc. (EA; Durham, NC) for globin transcript removal and whole transcriptome sequencing. Alpha- and beta-globin mRNA were substantially depleted from total RNA samples using a GlobinClear-Mouse/Rat kit (Applied Biosystems, Foster City, CA) (for details see [14]). The final globin mRNA-depleted RNA samples were quantitated by spectrophotometry using a NanoDrop ND-8000 (Thermo Scientific, Waltham, MA).

### mRNA quantitation

#### Transcript quantification in whole blood by RNA-seq profiling

Transcript expressions were determined by EA with their NGS sequencing methods, as previously described [14]. Briefly, EA used the globin mRNA-depleted RNA samples to create cDNA libraries using the TruSeq Stranded mRNA Sample Prep Kit (Illumina, San Diego, CA). Sequencing by synthesis methods, as implemented via Illumina technology, were used to generate the RNA-seq data. Fifty bp paired-end and strand-specific sequencing was performed on an Illumina platform generating 25–35 million reads per sample.

Quality control files contained read length and depth results (before and after clipping), presence of artifact/duplicate sequences, distribution of base quality, and base frequency by sample. Also, flow cell total yield, pass-filter reads, barcode quality, alignment summaries, and number of genes detected were determined. The basecall files were converted to fastq files using CASAVA 1.8.2. The fastq files were clipped using fastq-mcf with the parameters “—max-ns 4—qual-mean 25 -H -p 5 -q 7 -l 25” [39]. The fastq files were aligned to the rat Ensembl release 70 transcriptome (rn5) using bowtie 0.12.9 with the parameters “-e 500 –m 100 –chunkmbs 256”. The alignments were quantified using RSEM v 1.2.0 with no special parameters.

The RNA-seq data used in this study can be downloaded from NCBI GEO files GSE62368 and GSE64778. The IDs of the individual animals used in each group and assay are shown in Table 1. An additional set of samples was used to assess whether controls in a cool (16°C) environment responded similarly to controls in a 23°C environment. This set included three treatment groups: control (23°C environment), cool control (16°C environment), and AMPH hyper (S1 Table). There were no apparent differences in body temperature and expression profiles between the 16°C and 23°C control groups, including transcripts ascribed to organ damage or important immune-related genes (S1 and S2 Figs, and data not shown). Hence, all treatment comparisons, including of the AMPH normothermic group, were conducted relative to the 23°C control group.

**Table 1:** List of animal IDs included in mRNA and miRNA analyses.

	Animals used to determine RNA-seq profiles in whole blood		Animals used to determine the miRNA profiles in whole blood	
Treatment Group	Individual Animal IDs ^a^^,^^b^	Sample Size	Individual Animal IDs ^c^	Sample Size
**Control** **(saline given in a 23°C environment)**	ML322, JB108, FG82, FG88, FG89, FG93, FG94, FG98, FG99, FG135, FG136, FG150, FG151	13 (12)	ML322, FG82*, FG88*, FG89*, FG93*, FG98, FG135*, FG136*, FG150*, FG151*	**6** (10, 8*)
**AMPH normo (AMPH given in a 16°C environment)**	JB93, JB104, JB114, JB115, FG132, FG133, FG134, FG145, FG147, FG148, FG157, FG158, FG159	13 (13)	JB93, FG132*, FG134*, FG145*, FG147*, FG148*, FG157*, FG158*, FG159*	**6** (9, 8*)
**AMPH hyper (AMPH given in a 23°C environment)**	ML328, JB92, JB97, JB100, JB102, JB113, FG86, FG104, FG105, FG139, FG140, FG142, FG143, FG144	14 (12)	**ML328**, **JB100**, JB102*, FG86*, FG104*, FG105*, FG139*, FG140*, FG142*, FG143*, FG144*	**6** (11, 9*)
**EIH** **(saline given in a 39°C environment)**	ML324, JB90, JB109, JB110, FG90, FG91, FG92, FG95, FG97, FG100, FG101, FG102, FG137, FG138, FG152, FG153	16 (15)	**ML324, JB110**, FG90*, FG91*, FG92*, FG95*, FG97, FG100*, FG101*, FG137*, FG138*, FG152*, FG153*	**6** (13, 10*)

^a^ IDs are the same as listed in the GSE62368 and GSE64778 files

^b^ Underlined IDs, blood samples were used in follow-up RT-qPCR analyses. The remaining samples had insufficient RNA to be analyzed by RT-qPCR.

^c^ **Bold IDs**, blood samples used in TLDA and follow-up RT-qPCR analyses; IDs followed by *, serum samples used in RT-qPCR analyses. Serum samples were not available for all animals for which blood miRNAs were quantified by RT-qPCR, but the findings using all blood samples available or only the same sub-set of samples for which serum was also available were similar.

#### mRNA transcript quantification by quantitative reverse transcription–real-time polymerase chain reaction (RT-qPCR)

Reverse transcription of 500 ng of whole blood RNA was performed using random hexamers primers and SuperScript IV Reverse Transcriptase, followed by a digestion step at 37°C with *E*. *coli* RNase H (Invitrogen, Carlsbad, CA), following the manufacturer’s protocol. The cDNA was diluted 1:40 with nuclease-free water (Life Technologies, Foster City, CA) and used as a template in the qPCR assays. The expression of selected genes, identified as of interest based on the RNA-seq data, was quantified by qPCR in whole blood (n = 12-15/treatment group) using iQ SYBR Green Supermix and a CFX96 instrument (Bio-Rad Laboratories, Hercules, CA) and primers pairs designed using the NCBI Primer-BLAST software (S2 Table). The PCR cycling conditions were 1x 95°C for 10 min and 40x (95°C for 15 sec and 60°C for 1 min), followed by repetitive cycles of 5 sec starting at 65°C and incrementing in temperature by 0.5°C/cycle until 95°C to determine the melting curve data. Each primer pair yielded a single peak in the melting curve. Nuclease-free water (Life Technologies), instead of cDNA template, was added to the no-template control qPCR. Two independent qPCRs were performed to quantify the expression level of the endogenous control gene and the average of the two runs was used to normalize qPCR data.

### miRNA quantitation

#### RT-qPCR methods for miRNA analysis

cDNA was synthesized by using a TaqMan MicroRNA Reverse Transcription Kit (Life Technologies). Two-hundred nanograms of whole blood RNA or 3μL serum RNA were reverse-transcribed by using MegaPlex RT primers 10x, Rodent Pool A (Applied Biosystems) and MultiScribe™ Reverse Transcriptase, following the manufacturer’s instructions. RT product was pre-amplified (12 cycles) with MegaPlex™ PreAmp primers 10x (Applied Biosystems), according to the manufacturer’s protocol.

The expression level of 381 miRNAs was quantified by qPCR in whole blood (n = 6/treatment group) using TaqMan Low Density Arrays (TLDA Rodent microRNA Array A, Applied Biosystems). The TLDA cards were run in an ABI 7900HT Fast Real-Time PCR System (Applied Biosystems), following the manufacturer’s recommended qPCR cycling parameters. The amplification curves were analyzed using Sequence Detection Systems (SDS) software, version 2.4.1 (Applied Biosystems). A subset of miRNAs (n = 9) was selected based on the TLDA screening data and quantified by RT-qPCR in whole blood (n = 9-13/treatment group) and in available serum samples from the same animals (n = 8-10/treatment group). cDNA was synthesized and pre-amplified as described above and used as a template in qPCR reactions, which further included TaqMan Universal PCR Master Mix II, No AmpErase UNG, and a miRNA-specific TaqMan Small RNA assay (Life Technologies). Data were collected using a 7900HT instrument (Applied Biosystems), following the manufacturer’s protocol. S3 Table lists the TaqMan Small RNA assays used.

#### RNAseq methods for miRNA analysis

A total of 14 RNA samples (control = 7 and AMPH hyper = 7) was sent to EA genomics (http://www.q2labsolutions.com/genomics-laboratoriesR). Their RIN number ranged from 7.0 to 9.3, with the control values of 8.3 ± 0.7 and AMPH hyper values of 8.4 ± 0.8 (mean ± SD). Sequencing libraries were created using an adaptation of the Illumina TruSeq Small RNA method. Briefly, single stranded adenylated DNA adapters were added to the 3’-hydroxyl group of the sample RNAs using T4 RNA Ligase 2, deletion mutant enzyme. This enzyme prevents ligation of the adapter to the 5’ end due to the absence of ATP. 5’ adapters were then added to the 5’-phosphate using T4 RNA Ligase in the presence of ATP. Following adapter ligation, single stranded cDNA was created by a reverse transcription reaction. The cDNA was then PCR amplified for 14 cycles using a common sequencing primer and an indexed primer that is unique to each sample. The cDNA libraries were then size selected using the Pippin HT to ensure sequencing of small RNAs from 15–35 base pairs. The resulting libraries were quantified, normalized, and pooled in preparation for sequencing. Ten million reads per sample were generated from the libraries. The methods developed were designed to greatly enrich the microRNA (miRNA) portion of an RNA fraction. This size-specific enrichment allows for an enhanced ability to detect small non-coding RNAs and possibly degraded fragments of longer RNA.

### Data analysis

The RNA-seq by Expectation-Maximization (RSEM) [40] data generated by EA were rounded to produce count-based expression values. In total, 14,190 transcripts were included in the final analysis. Transcript-level outliers in each treatment group were identified by the iLOO [36]. and replaced with the trimmed mean. All reports of mean expression summarize DESeq2 normalized counts [41]. DESeq2 was also used to carry out differential expression analysis. Transcripts were deemed significant based on the following criteria: adjusted p-value (False Discovery Rate, FDR) < 0.05 and treatment mean must exceed DAFS global cutoff for low expression [14] or at least one of the two groups in pairwise comparison testing.

NormFinder [42] implemented in the R package NormqPCR [43] was used to identify the most stable miRNA from the TLDA screening data. NormFinder measures expression stability using model-based methods that account for both within and between-group variation. *miR-148a* was identified as the most stable miRNA and used as the endogenous control for miRNA data normalization. In previous work, *Rpl22* was identified as the most stably expressed transcript in mRNA expression data [14] and hence used as the endogenous control for normalization of the mRNA qPCR data. The relative levels of miRNAs and mRNAs in whole blood quantified by RT-qPCR were calculated using the comparative ΔΔCt method [44]. One-way ANOVA was used to evaluate the significance of five planned pairwise comparisons: AMPH normo, AMPH hyper, and EIH to control, AMPH hyper to AMPH normo, and AMPH hyper to EIH. An FDR-adjusted p-value < 0.05 was considered statistically significant. All analyses were carried out in R (http://www.r-project.org).

Pathway analysis was performed using the Ingenuity Pathway Analysis (IPA, Ingenuity Systems, Redwood City, CA) software v7.1, which uses Fisher’s exact test to determine the likelihood of an association between significantly expressed transcripts and pathways of interest being due to random chance. Fold-change data (treated versus control) were uploaded to IPA to identify significantly enriched signaling and metabolic canonical pathways in the current data based on the Ingenuity Knowledge Base. Genes with an FDR-adjusted p-value < 0.05 and a │fold-change│ ≥ 2 versus control were analyzed. Pathways were evaluated based on a p-value to assess the significance of overlap between molecules from the dataset and literature and a z-score to assess whether a pathway is activated (z-score≥2.0) or inhibited (z-score≤-2.0). Ranked results from top canonical pathways meeting: p-value<0.01,│z-score│≥2.0, and containing at least three focus molecules, were queried for functional annotations and over-representation to facilitate biological interpretation of selected gene lists.

## Results

### Body temperatures and survival

Approximately 5% to 10% of the treated animals in both the AMPH hyper and EIH groups were not used in the study because they were moribund, had blood glucose levels less than 25 mg/dL, or did not become sufficiently hyperthermic. The remaining animals were considered suitable for mRNA and miRNA analysis of whole blood, and over 85% of these animals would have been expected to have a long-term survival after their exposures. There were no overwhelming differences between body temperature profiles of the AMPH normothermic and control groups (S1 Fig). Both the EIH and AMPH hyper groups had higher body temperatures compared to the AMPH normothermic and control groups, but there were no apparent differences in the temperature profiles between the EIH and AMPH hyper groups. However, the AMPH hyper group needed from two to five cooling “sessions” (depending on the individual rat) to protect against lethal hyperthermia, while the EIH group required only one or two sessions. Thus, the body temperature of the AMPH hyper animals dropped below 40°C two to five times between the 3^rd^ and 9^th^ hr after the start of dosing, while this only occurred once or twice in the EIH group.

### RNA-seq differential expression analysis

Principal component analysis (PCA) was performed on ‘regularized log’ transformed RNA-seq data for the AMPH normo, AMPH hyper, EIH, control, and cool control treatment groups; the results are shown in S2 Fig. The cool control animals are surrounded by most of the main “core” of the control group. This suggests there is very little effect on the transcriptome due to a cool environment. It is also obvious that there are a few conspicuous outliers in several treatment groups. These outliers and the cool control group were removed and a PCA was subsequently performed on ‘regularized log’ transformed RNA-seq data for the AMPH normo, AMPH hyper, EIH, and control treatment groups.

A PCA score plot (3 principal components) is presented in Fig 1A. Treatment groups are summarized in S4 Table, where the mean and standard error of the DESeq2 normalized data are presented. A complete list of differentially expressed transcripts (i.e., FDR-adjusted p<0.05) for AMPH normo, AMPH hyper, and EIH relative to control is provided in S5 Table. The number of transcripts that were differentially expressed by at least 2-fold when comparing AMPH normo, AMPH hyper, and EIH to the control was: 483 (AMPH normo), 1180 (AMPH hyper), and 1591 (EIH) (Fig 1B). In addition, the list of differentially expressed transcripts between AMPH hyper relative to AMPH normo and EIH groups is provided in S6 Table. Of the differentially expressed transcripts, there were 820 and 182 differentially expressed transcripts with FC>2 when comparing AMPH hyper to AMPH normo and EIH, respectively.

**Fig 2:**
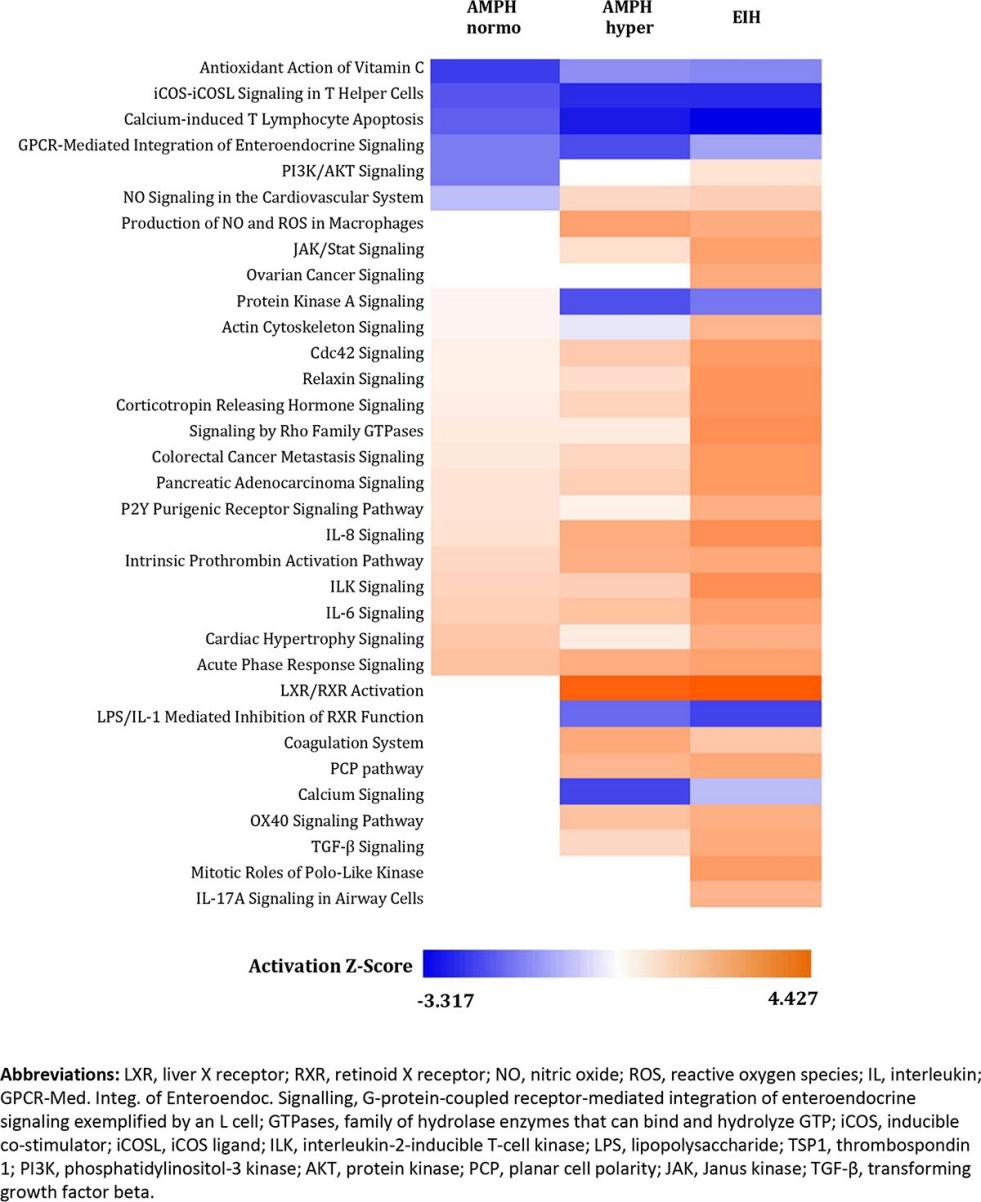
Heatmap displaying significant canonical pathways identified in the treatment groups of whole blood. The treatment groups AMPH normo, AMPH hyper, and EIH groups relative to control, and of AMPH hyper relative to AMPH normo and EIH were analyzed using the Fisher’s exact test at p<0.01. The color gradient reflects the predicted direction of change for each pathway, based on regulation z-score, where blue represents inhibited and orange represents activated.

Pathway analysis of the significant transcripts from the five pairwise comparisons was used to determine important changes produced by AMPH and/or hyperthermia (EIH). The canonical pathways significantly affected by treatment and the predicted direction of change for each pathway suggested a similar profile for the AMPH hyper and EIH groups (Fig 2). The number of pathways altered by the AMPH normo group relative to control was smaller than in the other two treatment groups. Moreover, their predicted activation/inhibition score tended to be smaller when compared to the controls (Fig 2 and S7 Table). Several pathways related to inflammation, including acute phase response and IL-6 and IL-8 signaling pathways, were activated in all treated groups relative to control. The LXR/RXR activation pathway was activated to a similar extent in AMPH hyper and EIH groups relative to control, while LPS/IL-1-mediated inhibition of RXR function pathway was inhibited in these two groups. Signaling pathways related to integrin-linked kinases and Rho GTPases, TFG-β, JAK/Stat, and actin cytoskeleton, all of which are involved in cell proliferation, migration, and apoptosis, were activated in the EIH group, and to a lesser extent in the AMPH hyper group, relative to control. In addition, the antioxidant action of vitamin C was inhibited, while the production of nitric oxide and reactive oxygen species in macrophages was activated (Fig 2 and S7 Table).

### White blood cell-specific mRNAs among treatment groups

**Fig 3:**
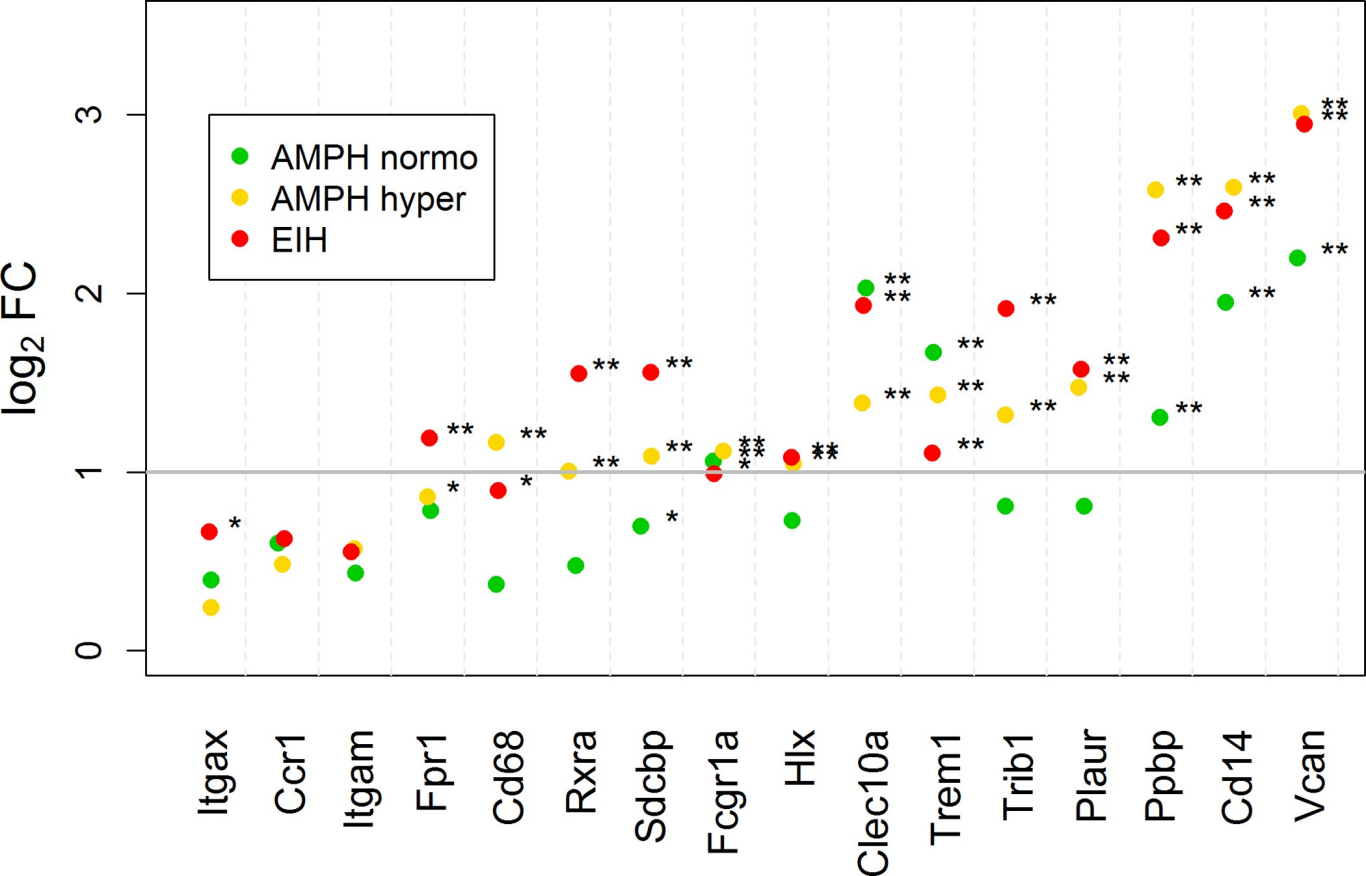
Scatterplot of fold-change expression for monocyte-specific mRNA transcripts in whole blood. The log2 fold-changes for the AMPH normo, AMPH hyper, and EIH groups relative to control are shown. The horizontal gray bar indicates a 2-fold increase in expression compared to control. The subscripts * and ** indicate that the fold-change is significant and exceeds 1.5 and 2, respectively.

**Fig 4:**
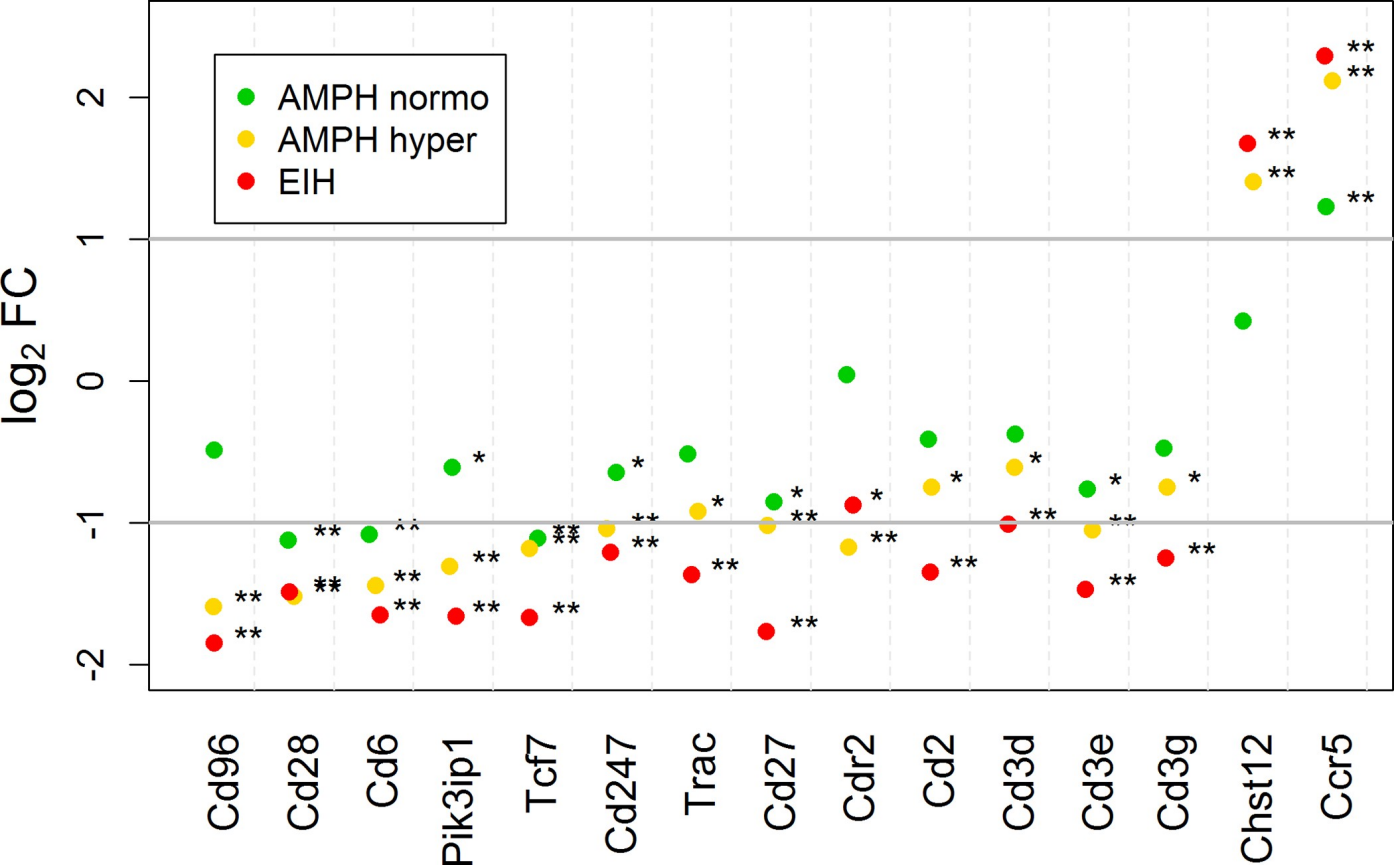
Scatterplot of fold-change expression for T-cell-specific mRNA transcripts in the whole blood. The log2 fold-change for the AMPH normo, AMPH hyper, and EIH groups relative to control are shown. The horizontal gray bars indicate a 2-fold increase and decrease in expression compared to control. The subscripts * and ** indicate that the fold-change is significant and exceeds 1.5 and 2, respectively.

**Fig 5:**
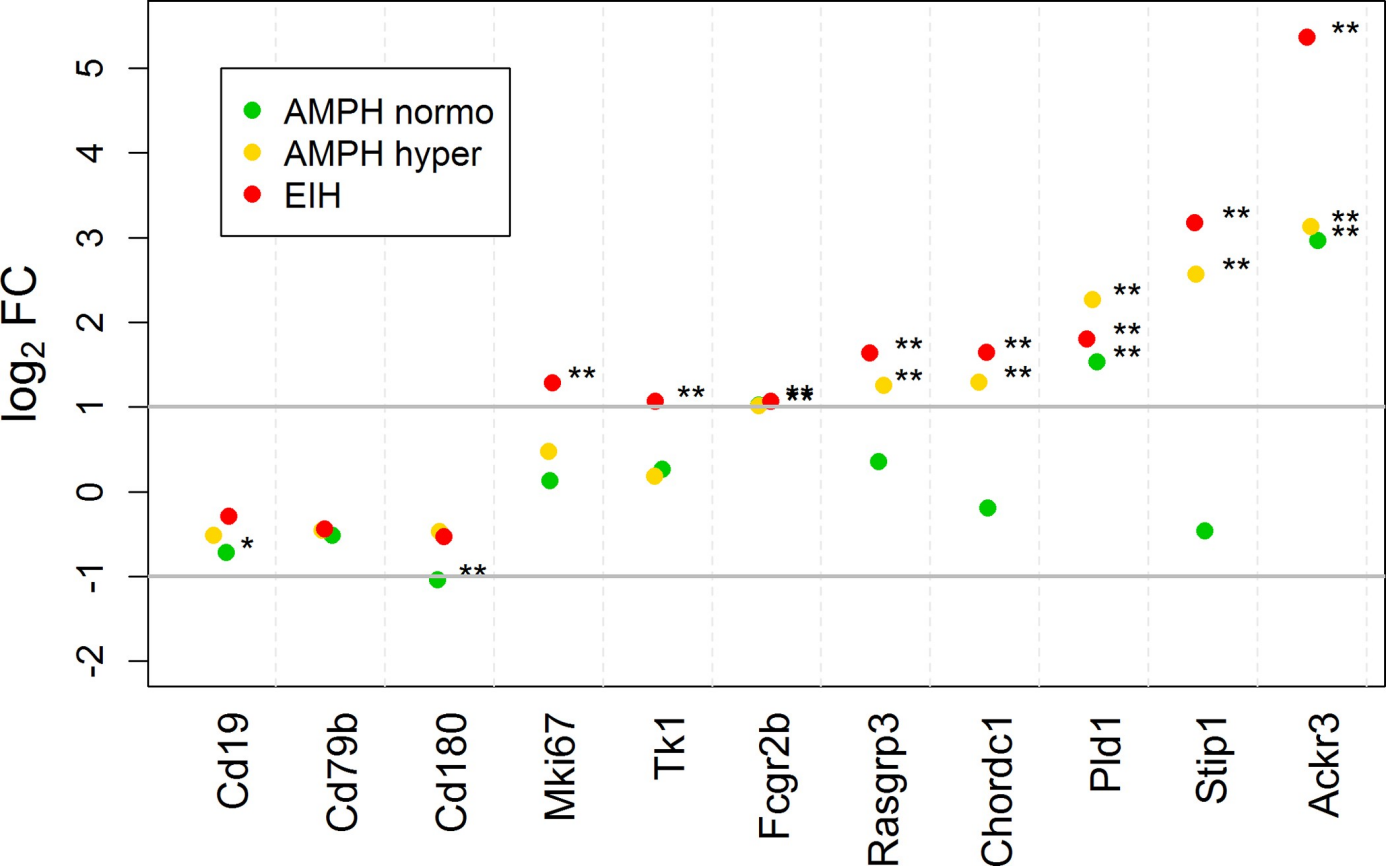
Scatterplot of fold-change expression for B-cell-specific mRNA transcripts in the whole blood. The log2 fold-change for the AMPH normo, AMPH hyper, and EIH groups relative to control. The horizontal gray bars indicate a 2-fold increase and decrease in expression compared to control. The subscripts * and ** indicate that the fold-change is significant and exceeds 1.5 and 2, respectively.

BioGPS [45] was used to determine the white blood cells (WBC) cell-type-specific genes shown in Fig 3 (monocytes), Fig 4 (T-cells), and Fig 5 (B-cells). Their origin was primarily determined using the available human and mouse transcriptomes. The transcriptome profiles in WBC cell-types for the mouse that are available in BioGPS appeared to correlate well with what was found in the corresponding human WBC cell-types. However, it is our opinion that the publicly available transcript expression references for blood or WBC cell-type in rats is incomplete at present, particularly compared to the human data. We hope these results will help build upon the current rat database for blood- and leukocyte-specific transcripts.

Fig 3 presents the RNAseq log2 FC expression of monocyte-specific transcripts for AMPH normo, AMPH hyper, and EIH relative to control. For many transcripts, AMPH hyper and EIH were elevated by more than 1.5-fold. This is expected in the AMPH hyper group because monocyte counts in the blood were approximately double relative to control [14]. It is likely that this is also true for EIH; however, leukocyte sub-types counts were not determined for the EIH treatment group. In the EIH and AMPH hyper groups, some transcripts, such as *Cd14*, *Clec10a*, and *Vcan*, increased in expression by more than 4-fold. This greater fold-increase is most likely due to an up-regulation of these genes in monocytes already present in the blood, monocytes that entering the blood having a much higher expression of these genes, or a combination of these two possibilities. Our data suggest that hyperthermia was not required for AMPH to increase monocyte-specific transcripts, since several transcripts were significantly expressed by 2- to 4-fold in the AMPH normo group. It was not determined to what extent leukocyte sub-types numbers changed in this group. Interestingly, *Clec10a* and *Trem1* were the only genes that increased to a greater extent in the AMPH normo group. Although not shown in Fig 3, the chemokine fractalkine receptor (*Cx3cr1*, found on monocytes, microglia, and maybe T-cells) paradoxically decreased in all three groups relative to control (S5 Table). RT-qPCR analysis confirmed the increases in fold-changes observed by RNA-seq for the monocyte membrane-associated transcript *Vcan* and *Cd14* for the AMPH groups (S8 Table). RT-qPCR analysis also demonstrated increased expression in these groups relative to control for *Ccr2*, which is another monocyte membrane-associated transcript, but to which there was no alignment in the rat Ensembl release 70 transcriptome (rn5).

Fig 4 shows the magnitude of expression in T-cell-specific transcripts, relative to control, for AMPH normo, AMPH hyper, and EIH. Nearly all, except two transcripts that are not exclusively expressed in T-cells (*Chst12* and *Ccr5*), were down-regulated by 2- to 4-fold in the AMPH hyper and EIH treatment groups. On the other hand, most transcripts specific to B-cells increased by 2- to 5-fold (Fig 5). Also, there was a more than 2-fold increase in immunoglobulin kappa constant (*Igkc*) and joining chain of multimeric IgA and IgM (*Jchain*) immunoglobulin transcripts in the AMPH hyper and EIH groups, but not in the AMPH normo group. These changes in both the T- and B-cells transcripts cannot be accounted for by changes in the blood cell count in the AMPH hyper group (and likely cannot for expression in the other two groups as well) (see Discussion). The reported fold-changes for *Cd3d*, *Cd3g*, *Ccr5*, and *Ackr3*, as determined by RT-qPCR, were similar to the fold-changes determined by RNA-seq (S8 Table).

### Tissue-specific transcripts in whole blood

To detect organ or tissue damage, the RNA-seq data were filtered for transcripts with very low expression in the control group and much higher levels in AMPH normo, AMPH hyper, and EIH. This method also identified some of the heat-shock and stress proteins expected to increase with hyperthermia. The BioGPS web site [45] was used to determine the tissue or organ specificity for the selected transcripts. This was determined by examining the human and mouse transcriptomes. Tissue and organ specificity for rat tissue transcriptome was not heavily relied upon because the data for transcript origins in rats are not complete, particularly with respect to blood.

The top 245 transcripts with the greatest fold-change in the AMPH hyper group relative to the control group generated the initial list of candidate transcripts (S9 Table; transcripts listed in descending fold-change in EIH group versus control). Transcripts were retained if they had an NCBI gene symbol and one or more of the groups had a treatment mean > 13 (DAFS cutoff) (S5 Table). Of the top 35 transcripts, 23 were specific for liver (S9 Table). In fact, over half of the transcripts in the list were specific for liver. Genes present only in pancreas (*Spink1*), bronchi/lungs (*Cyp4b1*), bone marrow (*Np4* [*PRTN3* is analog humans] and *RatNP-3b*), and kidney (*Cesl1* and *Slc22a8*) are also listed. Some of the genes present on the list are likely related to WBCs (e.g., *Ackr3)*, B-cells, or miscellaneous tissues (*Gpnmb*, *Ctsl1*, and *Gsg1*). Transcripts enriched in heart/skeletal muscle should be interpreted with caution, due to the possibility of an artifact due to collection of terminal blood by cardiac puncture. RT-qPCR validated our RNA-seq findings for the non-liver-specific genes, including *Ctsl1*, *F3*, *Gpnmb*, and *Gsg1* (S8 Table). However, the increased fold-changes in liver-specific transcripts (*Alb*, *Crp*, and *Fgb*) observed by RT-qPCR in the AMPH hyper group were smaller than those in the RNA-seq data, and not detected in the EIH group relative to control (S8 Table).

### miRNA profiles in whole blood and serum

Of the 381 miRNAs screened in whole blood by RT-qPCR, 26 miRNAs and two small nuclear RNAs were found differentially expressed in one or more treatment groups relative to control (S10 Table). Twenty of the 26 miRNAs were increased in whole blood by more than 2-fold in the AMPH normo group relative to control, of which seven were also increased by a similar fold in the AMPH hyper group. In contrast, three miRNAs were decreased in whole blood of the EIH group relative to control (S10 Table). There was a relatively good agreement between the RT-qPCR method and the RNAseq method of quantifying miRNA levels in blood with the RNAseq method showing a tendency for lower fold-changes in general (Table 2 and S10 and S11 Tables). However, the comparison between the RNAseq small RNA and RT-qPCR methods can only be made with the control and AMPH hyper groups. Some miRNAs that appeared to be affected in the AMPH hyper group compared to control when evaluated by the RNAseq method had not been evaluated with the RT-qPCR methods (S11 Table).

**Table 2:** Fold-change of selected miRNAs in the whole blood and serum of AMPH normo, AMPH hyper, and EIH groups relative to control.

	AMPH normo					AMPH hyper			EIH		
	Blood		Serum	Blood				Serum	Blood		Serum
miRNA	TLDA	qPCR	qPCR	TLDA	qPCR		RNAseq	qPCR	TLDA	qPCR	qPCR
*rno-miR-1-3p*	10.72*	7.27*	5.68	8.08*	8.19*		2.38	6.64	1.85	2.29	1.06
*mmu-miR-122-5p*	2.34	2.51	26.60	8.78	5.44*		-1.28	22.22	1.57	1.77	35.52
*mmu-miR-133a-3p*	15.26*	9.76*	4.62*	10.78*	9.60*		5.50*	4.44*	2.50	2.85	1.30
*mmu-miR-150-5p*	1.14	-1.05	2.26	-1.90	-1.36		-1.82	2.94*	-4.28*	-2.69*	1.73
*mmu-miR-204-5p*	4.43*	3.50*	1.54	2.45	2.43*		1.07	3.15	1.22	1.31	2.74
*mmu-miR-214-3p*	5.49*	5.29*	5.71*	4.53*	4.93*		2.78	3.65*	1.84	1.89	1.68
*mmu-miR-223-3p*	3.64*	2.41*	1.86	1.06	1.48		-1.15	-1.01	-1.88	-1.28	-1.55
*mmu-miR-375-3p*	2.79*	2.53*	6.10*	1.95	2.75*		1.78*	2.71	1.19	1.70	1.33
*mmu-miR-429-3p*	25.80*	2.86	ND	30.92*	15.6*		2.80*	ND	16.08	3.43	ND

^a^ ND, not detected

Fold changes were assessed by TLDA screening (whole blood), RNAseq (control and AMPH hyper whole blood only), and RT-qPCR (whole blood and serum). Data are presented as mean fold-change relative to control.

*, p<0.05.

Consistent with the findings of increased skeletal muscle/heart-enriched mRNA transcripts in AMPH normo and AMPH hyper groups relative to control, the levels of several miRNAs reported to be up-regulated upon skeletal muscle damage or cardiac toxicity, including *mmu-miR-1a-3p*, *rno-miR-1-3p*, *mmu-miR-133a-3p*, and *mmu-133b-3p*, were increased by more than 4-fold in the whole blood of AMPH-treated animals (Table 2). Follow-up RT-qPCR of *rno-miR-1-3p* and *mmu-miR-133a-3p* confirmed their increased levels in whole blood, as well as in serum, although this effect was not significant (Table 2). In addition, *mmu-miR-206-3p*, which belongs to the same miRNA family, but was not represented in the RT-qPCR array plate, was found not to be expressed in any of the controls, but was detected in 6 of the 7 AMPH hyper animals by the RNAseq method (S11 Table.

Given the increased levels of liver-specific transcripts, such as *Alb*, *Crp*, and *Fgb*, in whole blood and the well-documented increases of circulating *miR-122* upon liver injury, we quantified this miRNA in the whole blood and serum of study animals. Whole blood *mmu-miR-122-5p* was slightly increased by all treatments relative to control, with the effect being significant in the AMPH hyper group; however, a larger increase (> 22-fold) was observed in serum of all treated groups relative to control, although the large inter-animal variability resulted in non-statistical differences (Table 2). *mmu-miR-429-3p* was increased in blood by both AMPH exposures *versus* control; however, it was not detected in serum, suggesting that it is expressed in a blood cell rather than derived from a tissue (Table 2).

## Discussion

The objectives of this study were attained in that 1) non-hyperthermic- and hyperthermic-mediated effects of high AMPH exposure on immune system and tissue damage were evaluated based on mRNA and miRNA expression; 2) organ-specific transcripts and miRNAs that relate to damage produced by AMPH or EIH/heat stroke were identified; and 3) the initial changes in mRNA and miRNA transcripts that could affect infection susceptibility were identified from AMPH exposure and EIH/heat stroke. The new data relevant to the first objective are important for translating animal experiments to AMPH toxicity in humans. We have previously reported the effects of AMPH on blood transcriptomics when life-threatening hyperthermia occurs [14]; however, the translational relevance of the changes that occur by AMPH when animals remain normothermic is important. It is not known what percentage of AMPH and METH abusers experience significant hyperthermic events during their lifetime, and it is not a common occurrence during the majority of stimulant exposures that an abuser has over his or her lifetime. However, if it only happens once in an abuser’s life-time, a hyperthermic crisis would likely result in longer lasting neurotoxicity and at least acute liver damage from what is known in studies with laboratory animals [8, 12, 38, 46].

For the AMPH hyper group, the changes in the expression of leukocyte-specific genes present in whole blood during the initial/early period (3 h after the toxic exposure regimen) are only partially explained by increases in monocytes (2-fold) and decreases in the total combined numbers of B-cells and T-cells (30%) in the circulating blood [14]. Similar significant changes in leukocyte-specific genes also occurred in the AMPH normo and EIH groups; however, we did not assess sub-types of leukocytes for these two groups. Overall, the observed transcript changes and alterations in leukocyte numbers are the initial indicators for activation of the innate immune system. We did not detect a significant increase in *Il6* and *Tnf* in blood at the 3 hr time point, but they were also not observed to increase in meninges, choroid plexus, or brain tissues at this early time point in a previous study using the same toxic regimen [31]. However, at 24 h, both *Il6* and *Tnf* had increased 2- to 3- fold in these tissues [31]. Thus, following either environmental heat exposure or exposure to AMPH, some classic signs of innate immune system activation, as represented by *Il6* and *Tnf*, are present in laboratory animals.

We observed both an increased number of monocytes present in blood along with increased expression in cell surface proteins, such as *Cd14*, *Ccr2*, and *Vcan*, per monocyte present. The rapid rise in *Ccl2* and possibly *Ccl7* found in brain and other tissues produced by AMPH or METH [8, 19, 20, 22, 31] may be triggering this innate immune response through the Ccr2 receptors on monocytes and microglia [47–49]. In addition, damage-associated proteins from liver and other organs may be contributing to the innate immune response [50–53]. In conjunction with this, there is an apparent significant decrease in the number of circulating T-cells and possible down-regulation of some of their cell surface proteins per T-cell. This decrease may result from the rapid rise in *Ccl17*, a prime activator of T-cells [54, 55], transcripts in tissues such as brain, meninges and choroid plexus after neurotoxic exposures to either AMPH or METH [19, 20, 31]. Lastly, both an up-regulation and down-regulation of transcripts specific for B-cells was observed, which cannot be explained by changes in B-cell number in blood.

With respect to monocyte mRNA expression changes, the increases observed in *Ccr2* and possibly *Ccr5* would likely be expected to increase monocyte responsiveness to chemokines, while increases in *Vcan* and *Cd14* would be expected to increase the ability of monocytes to detect bacterial and viral components that produce monocyte activation [47]. The data indicate that the expression for all four of these genes was increased by AMPH under normothermic conditions. In addition, activation of *Trem1* amplifies monocyte-mediated inflammatory responses/activation triggered by bacterial and fungal infections by stimulating release of pro-inflammatory chemokines and cytokines. Its increase would be expected to result in monocyte activation and can result from AMPH exposure in the absence of hyperthermia. In contrast, unlike almost all the other monocyte transcripts, *Cx3cr1* mRNA paradoxically decreased in all three groups relative to control. The decrease in the fractalkine receptor would be expected to decrease monocyte activation; however, this receptor is also on T-cells and natural killer (NK) cells and may just reflect the decrease in the number of T-cells circulating in blood along with a decrease in the transcription of T-cell-specific genes.

Even if all the decrease in total lymphocytes were due to loss of T-cells, this would only amount to a 1.7-fold decrease in T-cell-specific transcripts. Hence, the most plausible explanation for the increased B-cell transcripts is through increased numbers of transcripts per B-cell. There were seemingly anomalous increases in the expression levels of several genes within the T-cells. The unexpected increase in *Ccr5* transcripts might be explained if it was up-regulated in the monocytes to overcome the lower levels of T-cells in blood. The unexpected increase in *Chst12* transcript might have occurred due to entry of NK cells into the circulation or an upregulation of this gene in NK cells, since the cells have very high transcript levels of this gene in humans [45]. The large fold-increases in *Pld1*, *Stip1*, and *Ackr3* in B-cells were significantly more than expected from any change in the number of B-cells in the blood.

The implications of a decrease in T-cells in the circulating blood, with a concomitant down-regulation of T-cell specific transcripts and an upregulation of B-cell transcript, are not clear. B-cells are known to be activated by T-cell-dependent and T-cell-independent mechanisms and it is possible that T-cell-independent B-cell activation is taking place following AMPH or hyperthermia. A fever-induced activation of B-cells could teleologically play a role in diseases in which high fever is produced, producing a polyclonal B-cell response. The upregulation of B-cell specific genes per B-cell would indicate an increase in B-cell activation, while the downregulation of T-cell-specific genes per T-cell would indicate a decrease. However, it is possible that many of the expression decreases in T-cell-specific genes are due to decreased numbers of T-cells within blood [14]. There were seemingly anomalous increases in the expression levels of several genes within the T- and B-cells. The more than doubling of immunoglobulin kappa constant (*Igkc*) and joining chain of multimeric IgA and IgM (*Jchain*) immunoglobulin transcripts in the AMPH hyper and EIH groups only indicates that extreme hyperthermia similar to heat stroke does activate B-cells.

In respect to T-cells, Saito *et al*. have previously reported the effects of METH on the immune status that have neurotoxic effects almost identical to AMPH in rodents [8, 38] in mice given a single 5 mg/kg dose or a ten day exposure to 5mg/kg per day [35]. Another mouse study by Martinez *et al*. used a similar single dose per day (but escalating by day with a 10 mg/kg ceiling) over a ten-day exposure, and observed signs of immunosuppression, reduced T-cell proliferation, and reduced resistance to fungal infection [56]. Some of the findings of the two mouse studies are similar to our results in rat. Saito *et al*. saw a decrease in the total number leukocytes of over 60% in mice, while our study saw leukocyte decreases ≤ 30% [14] and Martinez *et al*. observed signs of T-cell impairment. Our study results also indicated a significant down-regulation of genes important for T-cell immune function (*e*.*g*., *Cd3d*, *Cd3e*, and *Cd3ge*). Thus, all three studies saw signs of both very acute and more prolonged METH exposure adversely affecting T-cell function. There are significant differences between our study and the two mouse studies. Saito *et al*. saw a decrease in the NK cell activity acutely, while we found 4-fold-increase for the NK cell-specific transcript *Chst12* when AMPH produced hyperthermia or with heat stroke, indicating increases in NK cells. Some of these differences are surely due to the previous studies examining expression in later stages of innate immune system activation, while our study observed changes in the initial stages resulting from AMPH exposure. In addition, some differences may be due to our AMPH exposure being significantly more acutely intense and toxic.

*In vitro* studies suggest that METH enhances HIV-1 replication in various HIV-1–permissive cells, including monocyte-derived macrophages and dendritic cells [57, 58]. METH has also been shown to increase replication of the feline immunodeficiency virus in astrocytes. Intriguingly, the effects of METH on HIV-1 replication in human CD4^+^ T-cells that are the primary targets of HIV-1 infection and replication *in vivo* remain largely unclear [57]. Our study found increases in several transcripts associated with HIV that have been reported to exacerbate the severity of HIV and other viral infections, occurred during AMPH exposure even under normothermic conditions. Since the number of macrophages increased by 2-fold, the 5-fold-increase in *Ccr5*, a co-receptor of HIV, *in vivo* indicates a slightly greater than 2-fold-increase in the expression of *Ccr5*. This same effect was previously observed *in situ* in human primary monocyte cultures, where it was mediated by both dopamine D1 and D2 receptor stimulation [59, 60]. These results are commensurate with our *in vivo* observation that *Ccr5* expression can be upregulated by AMPH independent of hyperthermia. Also, our results indicate that hyperthermia alone, probably through activation of the innate immune system, can up-regulate *Ccr5* expression to an even greater extent than just dopamine receptor stimulation. *Ackr3*, *Pld1*, and *Ccr2* expression up-regulation had patterns very similar to *Ccr5* and further indicate that AMPH and EIH can exacerbate HIV-1 infection [61–63].

Many transcripts enriched in liver (e.g. *Alb*, *Crp*, and *Fgb*), which had little expression in the blood of controls and AMPH normo rats, were highly up-regulated in the AMPH hyper and EIH groups. This is due to hyperthermia damaging the liver [12, 13, 64], and almost certainly results from degenerating hepatocytes releasing mRNA into the circulating blood. The increase of serum miR-122 in these animals further suggests liver damage. Detection of up-regulated transcripts specific for pancreas (*Spink1*), bronchi/lungs (*Cyp4b1* and *F3*), and kidney (*Cesl1* and *Slc22a8*) indicate that damaged or degenerating cells from these organs are also releasing mRNA, but to a lesser extent. Such increases in mRNA specific for organ tissues was not detected in the AMPH normo group, which shows that this effect is related to hyperthermia produced by AMPH. It should be noted that the fold-changes observed in RT-qPCR data, especially with regards to liver-specific transcripts, were much lower than those observed in RNA-seq data for EIH and, to a lesser extent, AMPH hyper. This suggests that heat may cause modifications to the RNA molecules and interfere with the ability to detect them.

Some transcripts present in the whole blood of AMPH hyper and EIH rats, which were not contained within WBCs or RBCs, appear to be more likely to breakdown or undergo chemical reactions and/ or degradation so that they cannot be quantified using RT-qPCR. We postulate this because RNA-seq techniques can detect tremendous increases in the levels of several liver-specific transcripts (*Alb*, *Crp*, and Fgb), while these transcript elevations are less pronounced or not detected at all with RT-qPCR methods. This inability to quantify mRNA levels with RT-qPCR in the two hyperthermic group may be because their body temperature remained above 40°C for extended time periods, which will greatly increase tissue damage and reactive oxygen species (ROS) [8]. This could in turn increase the ability of the mRNA to react with constituents in the plasma and somehow interfere with their detection by RT-qPCR. This effect was greater in the EIH group than the AMPH hyper group, possibly because the EIH group had even longer continuous periods where the body temperature was above 40°C. At the miRNA level, the most changes detected by RT-qPCR were in the AMPH normo group, followed by the AMPH hyper group; however, it is perplexing that the RT-qPCR methods of quantification show the same or higher fold-changes when compared to the RNAseq method in several of the miRNAs affected as a result of the AMPH hyper exposure. Nonetheless, these results indicate that physiological factors such as hyperthermia may alter the ability to detect mRNA and miRNA in blood.

Several miRNAs were found modulated in the blood of AMPH-exposed animals *versus* the controls. The levels of *miR-1*, *miR-133a*, and *miR-206*, which play a role in skeletal muscle development, function, and disease [65], were increased by AMPH regardless of the thermic conditions. In addition, several miRNAs reported to affect aspects of immune and vascular function were also found increased, including *miR-429*, *miR-100*, and *miR-214*, [66–68]. Many of these miRNA changes were observed in both AMPH normo and AMPH hyper groups. Such changes may be related to regulation of leukocyte function.

### Conclusions

In this study the non-hyperthermic- and hyperthermic-mediated effects of a high dose AMPH were determined in the transcriptome of circulating blood. AMPH alone, in the absence of hyperthermia, was found capable of significantly affecting the immune status and leukocyte subtype-specific transcriptomics. The AMPH-dependent changes in leukocyte cell-type, along with the mRNA and miRNA changes that we observed, clearly manifest as early indicators of innate immune system activation in response to severe hyperthermia. Interestingly, extreme hyperthermia, like that producing heat-stroke, had very similar effects. The changes in leukocyte-specific transcripts also indicated that either AMPH (even when it does not produce hyperthermia) or extreme hyperthermia could increase the susceptibility to viral infection and/or accelerate the debilitating effects of viral infection. In addition, organ-specific transcripts that relate to damage produced by AMPH when it produces hyperthermia or EIH/heat stroke are present in blood acutely following exposure. This indicates that overt tissue damage that occurs during exposure to amphetamines is amplified greatly by hyperthermia.

## Supporting information

S1 Fig Time course of treatment effects on body temperature. This figure shows that rat body temperature is minimally affected by amphetamine (AMPH) when the room temperature is 16°C, but that severe hyperthermia usually occurs when AMPH is given and the room temperature is 23.5°C. It also shows that rats do not become hypothermic when given saline when the room temperature is 16°C.
(PDF)

S2 Fig PCA plot of all treatment groups. This PCA plot shows that the Cool Saline (given saline in a 16°C rather than 23.5°C normal room temperature) animal transcriptome in blood was virtually identical to the normal room temperature saline animals. The red symbols for the Cool Saline Controls are embedded and obscured among the black symbols representing the Saline Controls (room temperature). It also shows the outlier in the Saline Controls and other groups that were removed from analysis and are not present in Fig 1A shown in the manuscript.
(PDF)

S1 Table List of animal IDs in additional set. (DOCX)

S2 Table Primer pairs used for qPCR mRNA quantification assays. (DOCX)

S3 Table TaqMan assays used for qPCR miRNA quantification assays. (DOCX)

S4 Table Normalized expression levels for all gene transcripts returning at least one read for all four groups. (XLSX)

S5 Table List of genes in the three treatment groups that significantly differed from control group. 1^st^ Sheet: Genes differentially expressed between AMPH normo and Control at FDR-adj-p < 0.05; 2^nd^ Sheet: Genes differentially expressed between AMPH hyper and Control at FDR-adj-p < 0.05; 3^rd^ Sheet: Genes differentially expressed between EIH and Control at FDR-adj-p < 0.05.
(XLSX)

S6 Table List of genes that significantly differed between AMPH hyper versus AMPH normo or EIH groups. 1^st^ Sheet: Genes differentially expressed between AMPH hyper and AMPH normo at FDR-adj-p < 0.05; 2^nd^ Sheet: Genes differentially expressed between AMPH hyper and EIH at FDR-adj-p < 0.05.
(XLSX)

S7 Table Z-score and -log (p-value) for significantly modulated canonical pathways in the whole blood of AMPH normo, AMPH hyper, and EIH groups relative to control, and in AMPH hyper relative to AMPH normo and EIH. Only canonical pathways that had a p-value<0.01, │z-score│≥2. 0, and that contained at least three focus molecules for at least one of the treatment groups relative to control are listed.
(DOCX)

S8 Table Fold-change in mRNAs of whole blood from AMPH normo, AMPH hyper, and EIH relative to control, as assessed by RNA-seq and RT-qPCR. Data are presented as mean fold-change relative to control. *, p<0.05.
(DOCX)

S9 Table The top 245 transcripts with the greatest fold-change in the AMPH hyper group relative to the control group. (XLSX)

S10 Table Fold-change in miRNAs in the whole blood of AMPH normo, AMPH hyper, and EIH groups relative to control, as assessed by TLDA cards or RNAseq (AMPH hyper only). Data are presented as mean fold-change relative to control. * p<0.05.
(DOCX)

S11 Table List of mature miRNA detected by small RNAseq techniques. (DOCX)
