# Supplementary figures and images for: Identification of whole blood mRNA and microRNA biomarkers of tissue damage and immune function resulting from amphetamine exposure or heat stroke in adult male rats
Source: PLoS One. 2019 Feb 19;14(2):e0210273. doi: 10.1371/journal.pone.0210273 (PMC6380594; doi:10.1371/journal.pone.0210273)

S1 Fig.

## Time Course of Treatment Effects on Body Temperature

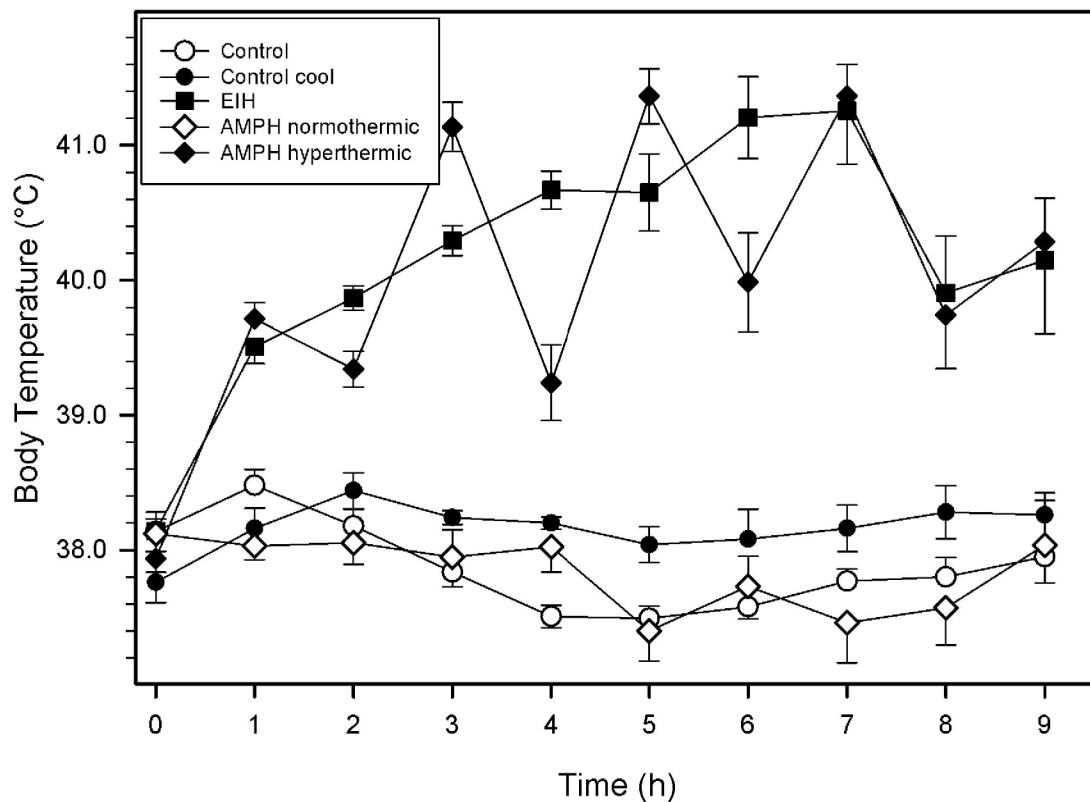

Supplement: S1 Fig — This figure shows that rat body temperature is minimally affected by amphetamine (AMPH) when the room temperature is 16°C, but that severe hyperthermia usually occurs when AMPH is given and the room temperature is 23.5°C. It also shows that rats do not become hypothermic when given saline when the room temperature is 16°C. (PDF) [file pone.0210273.s001.pdf]

S2 Fig.  
PCA plot of all treatment groups.

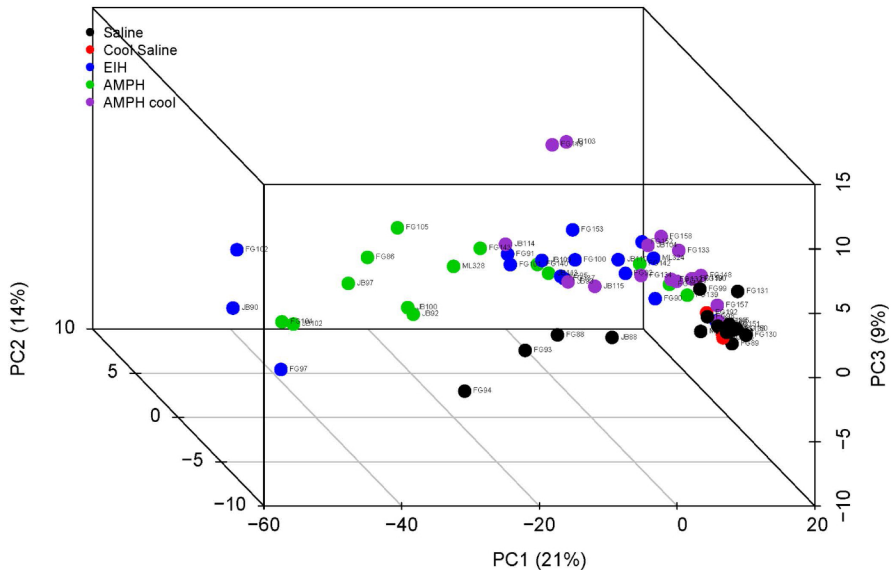

Supplement: S2 Fig — This PCA plot shows that the Cool Saline (given saline in a 16°C rather than 23.5°C normal room temperature) animal transcriptome in blood was virtually identical to the normal room temperature saline animals. The red symbols for the Cool Saline Controls are embedded and obscured among the black symbols representing the Saline Controls (room temperature). It also shows the outlier in the Saline Controls and other groups that were removed from analysis and are not present in Fig 1A shown in the manuscript. (PDF) [file pone.0210273.s002.pdf]
