# Supplementary material for: Identification of whole blood mRNA and microRNA biomarkers of tissue damage and immune function resulting from amphetamine exposure or heat stroke in adult male rats
Source: PLoS One. 2019 Feb 19;14(2):e0210273. doi: 10.1371/journal.pone.0210273 (PMC6380594; doi:10.1371/journal.pone.0210273)
Supplement: S1 Table — (DOCX) [file pone.0210273.s003.docx]

**S1** **Table. List of animal IDs in additional set.**

| **Treatment Group** | **Individual Animal IDs ^a^** |
| --- | --- |
| **Cool control  (saline given in a 16°C environment)** | FG190, FG191, FG192, FG193, FG194 |
| **Control  (saline given in a 23°C environment)** | FG180W, FG181W, FG196W, FG200W, FG201W, FG202W, |
| **AMPH hyper**  **(AMPH given in a 23°C environment)** | FG184W, FG185W, FG188W, FG189W, FG197W, FG198W, FG199W, FG204W, |

^a^ IDs are the same as listed in the GSE64778 file
