# Supplementary material for: Identification of whole blood mRNA and microRNA biomarkers of tissue damage and immune function resulting from amphetamine exposure or heat stroke in adult male rats
Source: PLoS One. 2019 Feb 19;14(2):e0210273. doi: 10.1371/journal.pone.0210273 (PMC6380594; doi:10.1371/journal.pone.0210273)
Supplement: S2 Table — (DOCX) [file pone.0210273.s004.docx]

**S2** **Table. Primer pairs used for qPCR mRNA quantification assays.**

| Gene Symbol | **Gene ID** | **Direction** | **Primer Sequence (5’ → 3’)** | **Amplicon Length (bp)** | **Mean Ct (Control)** |
| --- | --- | --- | --- | --- | --- |
| *Ackr3* | XM_006245479.3 | Forward | CAGCACTCAAAGCCAGGAAG | 294 | 33.47 |
|  |  | Reverse | GCTACTGTTACAGGGCCAGTT |  |  |
| *Alb* | NM_134326.2 | Forward | CTGCCGATCTGCCCTCAATA | 98 | 29.17 |
|  |  | Reverse | CAAAAACGTGCCCAGGAAGAC |  |  |
| *Ccr2* | NM_021866.1 | Forward | CCTTTGTTGGTGAGAAGTTCCG | 121 | 30.34 |
|  |  | Reverse | GTTGAGCTCACTCGGTCTGC |  |  |
| *Ccr5* | NM_053960.3 | Forward | GAGCTGGGCTGCAATTTGTT | 126 | 30.30 |
|  |  | Reverse | ACAAGAAACTCTGGCTCTTGC |  |  |
| *Cd3d* | NM_013169.1 | Forward | CACTCATCTTGGGCAAAGGC | 121 | 28.46 |
|  |  | Reverse | ACACAGTTCTGGCACATTCG |  |  |
| *Cd3g* | NM_001077646.2 | Forward | CTCTCTTCTTCAAGGCACCA | 104 | 26.84 |
|  |  | Reverse | TGAAGTCACAAGTCAGAAGTACAG |  |  |
| *Cd14* | NM_021744.1 | Forward | CTCAGAATCTACCGACCATGAAGC | 101 | 30.16 |
|  |  | Reverse | GTCCAGCTCACAGGGTTCTG |  |  |
| *Crp* | NM_017096.3 | Forward | GTGGCTTTGACGCGAATCAG | 117 | 29.24 |
|  |  | Reverse | TGGGGCTGAATACCCTACCA |  |  |
| *Ctsl1* | NM_013156.2 | Forward | CTCTGCTTGGGAACAGCCTT | 113 | 30.40 |
