# Supplementary material for: Identification of whole blood mRNA and microRNA biomarkers of tissue damage and immune function resulting from amphetamine exposure or heat stroke in adult male rats
Source: PLoS One. 2019 Feb 19;14(2):e0210273. doi: 10.1371/journal.pone.0210273 (PMC6380594; doi:10.1371/journal.pone.0210273)
Supplement: S3 Table — (DOCX) [file pone.0210273.s005.docx]

**S3 Table. TaqMan assays used for qPCR miRNA quantification assays.**

| **miRNA Name** | **miRBase Accession Number** | **Assay ID** |
| --- | --- | --- |
| *hsa-miR-133a* | MIMAT0000427 | 002246 |
| *hsa-miR-148a* | MIMAT0000243 | 000470 |
| *mmu-miR-122* | MIMAT0000421 | 002245 |
| *mmu-miR-150* | MIMAT0000451 | 000473 |
| *mmu-miR-204* | MIMAT0000265 | 000508 |
| *mmu-miR-214* | MIMAT0000271 | 002306 |
| *mmu-miR-223* | MIMAT0000280 | 002295 |
| *mmu-miR-375* | MIMAT0000728 | 000564 |
| *mmu-miR-429* | MIMAT0001537 | 001077 |
| *rno-miR-1* | MIMAT0003125 | 002064 |
