# Supplementary material for: Identification of whole blood mRNA and microRNA biomarkers of tissue damage and immune function resulting from amphetamine exposure or heat stroke in adult male rats
Source: PLoS One. 2019 Feb 19;14(2):e0210273. doi: 10.1371/journal.pone.0210273 (PMC6380594; doi:10.1371/journal.pone.0210273)
Supplement: S7 Table — Only canonical pathways that had a p-value<0.01, │z-score│≥2. 0, and that contained at least three focus molecules for at least one of the treatment groups relative to control are listed. (DOCX) [file pone.0210273.s009.docx]

**S7 Table. Z-score and -log (p-value) for significantly modulated canonical pathways in the whole blood of AMPH normo, AMPH hyper, and EIH groups relative to control, and in AMPH hyper relative to AMPH normo and EIH.** Only canonical pathways that had a p-value<0.01, │z-score│≥2. 0, and that contained at least three focus molecules for at least one of the treatment groups relative to control are listed.

| **Canonical Pathway** | **AMPH normo**  **vs control** | | **AMPH hyper**  **vs control** | | **EIH**  **vs control** | |
| --- | --- | --- | --- | --- | --- | --- |
|  | **Z-score** | **-log**  **(p-value)** | **Z-score** | **-log**  **(p-value)** | **Z-score** | **-log**  **(p-value)** |
| LXR/RXR Activation | NaN^a^ | 0.99 | 4.22 | 19.99 | 4.43 | 16.31 |
| Calcium-induced T Lymphocyte Apoptosis | -2.00 | 1.95 | -2.89 | 3.81 | -3.21 | 3.72 |
| Acute Phase Response Signaling | 1.63 | 1.21 | 2.20 | 15.13 | 2.50 | 16.29 |
| IL-8 Signaling | 0.77 | 4.85 | 2.18 | 1.77 | 2.98 | 0.95 |
| Intrinsic Prothrombin Activation Pathway | 1.00 | 2.79 | 2.12 | 3.55 | 2.31 | 6.18 |
| Production of NO and ROS in Macrophages | 0.00 | 1.72 | 2.50 | 5.40 | 2.19 | 4.31 |
| iCOS-iCOSL Signaling in T Helper Cells | -2.12 | 3.01 | -2.67 | 3.59 | -2.68 | 5.79 |
| ILK Signaling | 1.15 | 3.10 | 1.29 | 1.24 | 2.98 | 1.87 |
| IL-6 Signaling | 1.26 | 4.18 | 1.60 | 2.51 | 2.50 | 2.02 |
| Signaling by Rho Family GTPases | 0.53 | 3.63 | 0.53 | 0.59 | 2.98 | 1.43 |
| Pancreatic Adenocarcinoma Signaling | 0.71 | 3.08 | 1.26 | 2.36 | 2.71 | 1.62 |
| LPS/IL-1 Mediated Inhibition of RXR Function | NaN | 2.66 | -1.89 | 12.36 | -2.36 | 14.51 |
| Protein Kinase A Signaling | 0.28 | 3.61 | -2.20 | 2.11 | -1.71 | 3.28 |
| Antioxidant Action of Vitamin C | -2.45 | 2.17 | -1.41 | 1.56 | -1.51 | 1.36 |
| Corticotropin Releasing Hormone Signaling | 0.45 | 1.61 | 1.15 | 2.97 | 2.83 | 3.47 |
| Cardiac Hypertrophy Signaling | 1.51 | 2.01 | 0.50 | 0.74 | 2.13 | 0.92 |
| Cdc42 Signaling | 0.38 | 1.25 | 1.41 | 0.98 | 2.67 | 2.74 |
| NO Signaling in the Cardiovascular System | -0.82 | 1.54 | 1.00 | 3.54 | 1.29 | 1.84 |
| GPCR-Mediated Integration of Enteroendocrine Signaling Exemplified by an L Cell | 1.63 | 2.42 | -2.24 | 0.47 | -1.13 | 0.58 |
| Coagulation System | NaN | 0.79 | 2.32 | 10.12 | 1.50 | 9.34 |
| P2Y Purigenic Receptor Signaling Pathway | 0.71 | 2.19 | 0.33 | 0.56 | 2.14 | 1.01 |
| Colorectal Cancer Metastasis Signaling | 0.58 | 2.72 | 1.09 | 1.91 | 2.71 | 1.29 |
| PI3K/AKT Signaling | -1.63 | 2.37 | 0.00 | 1.18 | 0.71 | 0.40 |
| PCP pathway | NaN | 0.00 | 2.00 | 0.39 | 2.33 | 1.55 |
| Calcium Signaling | NaN | 2.94 | -2.33 | 1.10 | -0.83 | 1.92 |
| TGF-β Signaling | 0.38 | 0.99 | 0.90 | 2.70 | 2.84 | 0.55 |
| Mitotic Roles of Polo-Like Kinase | 0.28 | 0.41 | -0.30 | 0.57 | 2.00 | 1.36 |
| Ovarian Cancer Signaling | NaN | 2.01 | 1.63 | 0.46 | 2.12 | 0.64 |
| IL-17A Signaling in Airway Cells | 0.00 | 0.67 | 0.82 | 0.00 | 2.53 | 0.00 |
| Relaxin Signaling | NaN | 2.29 | 1.00 | 1.72 | 2.24 | 2.75 |
| Actin Cytoskeleton Signaling | NaN | 3.58 | NaN | 0.37 | 2.65 | 0.87 |
| OX40 Signaling Pathway | 0.00 | 0.25 | NaN | 1.67 | 2.24 | 3.18 |
| JAK/Stat Signaling | NaN | 2.15 | NaN | 0.56 | 2.00 | 1.15 |

^a^ NaN, undefined value. Z-score could not be calculated, since less than four genes were affected in the canonical pathway. Other abbreviations defined in Fig 2.
