# Supplementary material for: Identification of whole blood mRNA and microRNA biomarkers of tissue damage and immune function resulting from amphetamine exposure or heat stroke in adult male rats
Source: PLoS One. 2019 Feb 19;14(2):e0210273. doi: 10.1371/journal.pone.0210273 (PMC6380594; doi:10.1371/journal.pone.0210273)
Supplement: S8 Table — Data are presented as mean fold-change relative to control. *, p<0.05. (DOCX) [file pone.0210273.s010.docx]

**S8 Table.** **Fold-change in mRNAs of whole blood from AMPH normo, AMPH hyper, and EIH relative to control, as assessed by RNA-seq and RT-qPCR.** Data are presented as mean fold-change relative to control. *, p<0.05.

|  | **AMPH normo** | | **AMPH hyper** | | **EIH** | |
| --- | --- | --- | --- | --- | --- | --- |
| **Gene Symbol** | **RNA-seq** | **qPCR** | **RNA-seq** | **qPCR** | **RNA-seq** | **qPCR** |
| *Ackr3* | 7.81* | 4.21* | 8.78* | 6.19* | 41.22* | 7.44* |
| *Alb* | -1.50* | 1.61 | 34.48* | 5.91* | 53.32* | 1.31 |
| *Ccr2* | ND | 2.61* | ND | 4.44* | ND | 2.79 |
| *Ccr5* | 2.34* | 1.89 | 4.33* | 5.01* | 4.89* | 2.69 |
| *Cd3d* | -1.29* | -2.33* | -1.52* | -2.40* | -2.01* | -4.27* |
| *Cd3g* | -1.39* | -2.73* | -1.68* | -2.74* | -2.37* | -4.84* |
| *Cd14* | 3.87* | 2.93* | 6.04* | 5.52* | 5.51* | 3.63 |
| *Crp* | -1.24* | 2.61 | 17.31* | 2.85 | 43.52* | 1.52 |
| *Ctsl1* | 2.01* | 1.38 | 6.41* | 4.72* | 7.06* | 3.28 |
| *F3* | 1.79* | 1.67 | 13.41* | 5.39* | 30.94* | 7.39* |
| *Fgb* | 10.28* | 2.49 | 345.47* | 2.01 | 401.84* | 1.73 |
| *Gpnmb* | 2.06* | 1.72* | 5.27* | 3.68* | 10.42* | 6.43* |
| *Gsg1* | 3.31* | 2.35* | 19.97* | 12.04* | 12.33* | 5.58* |
| *Vcan* | 4.59* | 3.78* | 8.05* | 5.31* | 7.72* | 3.32 |

ND, not determined since *Ccr2* transcript was not aligned for in the rat Ensembl release 70 transcriptome (rn5).
