# Supplementary material for: Identification of whole blood mRNA and microRNA biomarkers of tissue damage and immune function resulting from amphetamine exposure or heat stroke in adult male rats
Source: PLoS One. 2019 Feb 19;14(2):e0210273. doi: 10.1371/journal.pone.0210273 (PMC6380594; doi:10.1371/journal.pone.0210273)
Supplement: S10 Table — Data are presented as mean fold-change relative to control. * p<0.05. (DOCX) [file pone.0210273.s012.docx]

**S10 Table. Fold-change in miRNAs in the whole blood of AMPH normo, AMPH hyper, and EIH groups relative to control, as assessed by TLDA cards or RNAseq (AMPH hyper only).** Data are presented as mean fold-change relative to control. * p<0.05.

|  | **AMPH normo** | **AMPH hyper** | | **EIH** |
| --- | --- | --- | --- | --- |
| **miRNA** | **qPCR** | **qPCR** | **RNAseq** | **qPCR** |
| *rno-miR-1-3p^a^* | 10.72* | 8.08* | 2.38* | 1.85 |
| *mmu-miR-1a-3p* | 4.75* | 5.39* | 3.66 | 1.49 |
| *mmu-miR-100-5p* | 2.31 | 2.44* | 1.42 | 1.51 |
| *mmu-miR-125a-5p* | 3.01* | 1.03 | 1.15 | -1.14 |
| *mmu-miR-126a-3p* | 2.52* | 1.08 | -1.12 | -1.63 |
| *mmu-miR-126a-5p* | 2.43* | 1.32 | -1.35 | -1.11 |
| *mmu-miR-133a-3p^a^* | 15.26* | 10.78* | 5.50* | 2.50 |
| *mmu-miR-133b-3p* | 12.94* | 18.71* | 3.25 | 5.19 |
| *mmu-miR-139-5p* | 1.18 | -2.38 | -1.23 | -4.87* |
| *mmu-miR-146b-5p* | 2.01* | -1.02 | 1.05 | -1.39 |
| *mmu-miR-150-5p^a^* | 1.14 | -1.90 | -1.82 | -4.28* |
| *mmu-miR-152-3p* | 2.32* | 1.73 | 1.91 | -1.15 |
| *mmu-miR-191-5p* | 2.34* | -1.37 | -1.10 | -1.48 |
| *mmu-miR-192-5p* | 2.03* | 1.36 | -1.08 | -1.52 |
| *mmu-miR-199a-3p* | 4.18* | 2.77* | 1.58 | 1.13 |
| *mmu-miR-203-3p* | 2.76* | 1.32 | -1.10 | -1.16 |
| *mmu-miR-204-5p^a^* | 4.43* | 2.45 | 1.07 | 1.22 |
| *mmu-miR-205-5p* | 9.30* | 3.25 | 3.03 | 1.53 |
| *mmu-miR-214-3p^a^* | 5.49* | 4.53* | 2.78 | 1.84 |
| *mmu-miR-223-3p^a^* | 3.64* | 1.06 | 1.15 | -1.88 |
| *mmu-miR-375-3p^a^* | 2.79* | 1.95 | 1.78* | 1.19 |
| *mmu-miR-429-3p^a^* | 25.80* | 30.92* | 2.80* | 16.08 |
| *mmu-miR-542-3p* | -1.13 | -1.97 | -1.33 | -2.13* |
| *mmu-miR-574-3p* | 2.70* | 1.18 | - | -1.38 |
| *mmu-miR-685* | 1.20 | 4.81* | - | 1.22 |
| *snoRNA135* | -1.06 | -2.12 | - | -3.37* |
| *U87* | 1.22 | -1.49 | - | -3.87* |

*^a^*miRNAs were further analyzed by RT-qPCR (data in Table 2); -, no RNAseq data available
