# Supplementary material for: Identification of whole blood mRNA and microRNA biomarkers of tissue damage and immune function resulting from amphetamine exposure or heat stroke in adult male rats
Source: PLoS One. 2019 Feb 19;14(2):e0210273. doi: 10.1371/journal.pone.0210273 (PMC6380594; doi:10.1371/journal.pone.0210273)
Supplement: S11 Table — (DOCX) [file pone.0210273.s013.docx]

**S11 Table. List of mature miRNA detected by small RNAseq techniques.**

**Color legend:**

|  | miRNAs found in humans in serum, exosomes, or RBCs published in Juzenas *et al.* (2017) in Figure 2 | | | | |
| --- | --- | --- | --- | --- | --- |
|  | miRNAs not found in human RBCs published in Juzenas *et al.* (2017), Figure 2 | | | |  |
|  | miRNAs that may come from damaged tissue | | | |  |
|  | miRNAs that may or may not be mentioned in published in Juzenas *et al.* (2017), Figure 2 | | | |  |
|  | |  |  |  |  |
| **miRNA ID** | | **Control**  **average count** | **AMPH hyper average count** |  |  |
| rno-miR-486 | | 21447490.6 | 24977699.4 |  |  |
| rno-miR-16-5p | | 4014483.9 | 3684192.7 |  |  |
| rno-miR-26a-5p | | 2630106.1 | 2582864.9 |  |  |
| rno-miR-191a-5p | | 1750887.6 | 1678230.2 |  |  |
| rno-let-7f-5p | | 1225386.7 | 1247569.6 |  |  |
| rno-let-7i-5p | | 970242.5 | 1051715.5 |  |  |
| rno-miR-181a-5p | | 775496.1 | 728699.0 |  |  |
| rno-miR-451-5p | | 604281.8 | 615332.3 |  |  |
| rno-miR-25-3p | | 558888.8 | 539907.4 |  |  |
| rno-miR-27b-3p | | 547795.1 | 514698.7 |  |  |
| rno-let-7a-5p | | 489774.2 | 581177.7 |  |  |
| rno-let-7c-5p | | 427361.2 | 498485.8 |  |  |
| rno-miR-144-5p | | 398901.1 | 369406.3 |  |  |
| rno-miR-22-3p | | 360431.6 | 335999.3 |  |  |
| rno-miR-144-3p | | 336673.7 | 281011.4 |  |  |
| rno-miR-142-5p | | 327524.2 | 216119.7 |  |  |
| rno-let-7g-5p | | 291995.0 | 296424.9 |  |  |
| rno-miR-186-5p | | 267246.8 | 269125.2 |  |  |
| rno-miR-93-5p | | 211434.3 | 203234.2 |  |  |
| rno-let-7d-5p | | 163150.5 | 192404.5 |  |  |
| rno-miR-301a-3p | | 123933.7 | 104518.0 |  |  |
| rno-miR-103-3p | | 111438.3 | 117270.3 |  |  |
| rno-miR-92a-3p | | 99541.8 | 119601.0 |  |  |
| rno-miR-21-5p | | 95944.8 | 78493.2 |  |  |
| rno-let-7b-5p | | 92663.7 | 107203.7 |  |  |
| rno-miR-181c-5p | | 89729.3 | 101094.3 |  |  |
| rno-miR-151-3p | | 85763.2 | 90546.9 |  |  |
| rno-miR-15b-5p | | 82625.4 | 84005.8 |  |  |
| rno-miR-30a-5p | | 69933.4 | 70740.3 |  |  |
| rno-miR-143-3p | | 61012.9 | 61217.0 |  |  |
| rno-miR-30d-5p | | 59912.0 | 63166.5 |  |  |
| rno-miR-98-5p | | 59557.8 | 61783.5 |  |  |
| rno-miR-17-5p | | 53819.2 | 55192.4 |  |  |
| rno-miR-19b-3p | | 46801.6 | 41595.2 |  |  |
| rno-miR-423-3p | | 44972.4 | 54148.8 |  |  |
| rno-miR-150-5p | | 41935.9 | 28797.1 |  |  |
| rno-miR-20a-5p | | 40903.5 | 43689.9 |  |  |
| rno-miR-192-5p | | 40019.7 | 37558.1 |  |  |
| rno-miR-146b-5p | | 38187.8 | 41029.7 |  |  |
| rno-miR-142-3p | | 36844.2 | 37860.0 |  |  |
| rno-miR-26b-5p | | 36240.1 | 30927.2 |  |  |
| rno-miR-425-5p | | 32694.0 | 35577.0 |  |  |
| rno-miR-378a-3p | | 31603.5 | 34249.8 |  |  |
| rno-miR-181b-5p | | 30116.1 | 33212.2 |  |  |
| rno-miR-128-3p | | 29772.1 | 29320.0 |  |  |
| rno-miR-30b-5p | | 29378.7 | 30566.9 |  |  |
| rno-miR-151-5p | | 28716.7 | 29208.5 |  |  |
| rno-miR-106b-5p | | 27411.3 | 23827.2 |  |  |
| rno-miR-320-3p | | 25154.1 | 29181.5 |  |  |
| rno-miR-30c-5p | | 24369.4 | 23754.0 |  |  |
| rno-miR-3557-5p | | 24079.7 | 25969.9 |  |  |
| rno-miR-106b-3p | | 23948.4 | 24726.2 |  |  |
| rno-miR-340-5p | | 22777.6 | 24681.8 |  |  |
| rno-miR-101a-3p | | 21257.8 | 20180.3 |  |  |
| rno-miR-181d-5p | | 20539.6 | 21907.9 |  |  |
| rno-miR-10a-5p | | 19811.5 | 35171.3 |  |  |
| rno-miR-223-3p | | 19138.0 | 16817.3 |  |  |
| rno-let-7d-3p | | 18777.3 | 21636.9 |  |  |
| rno-miR-27a-3p | | 17074.4 | 15596.9 |  |  |
| rno-miR-30e-5p | | 14817.2 | 12943.4 |  |  |
| rno-miR-423-5p | | 14532.3 | 13859.4 |  |  |
| rno-miR-148a-5p | | 13086.1 | 11387.1 |  |  |
| rno-miR-29a-3p | | 12125.3 | 11225.1 |  |  |
| rno-miR-3586-3p | | 11272.7 | 11375.6 |  |  |
| rno-miR-107-3p | | 11169.1 | 12692.6 |  |  |
| rno-miR-30e-3p | | 10663.8 | 9864.8 |  |  |
| rno-miR-19a-3p | | 9522.9 | 8322.0 |  |  |
| rno-miR-484 | | 9484.9 | 9800.4 |  |  |
| rno-miR-126a-5p | | 9470.3 | 7113.7 |  |  |
| rno-miR-210-3p | | 8782.5 | 9677.8 |  |  |
| rno-miR-18a-5p | | 8364.3 | 8730.4 |  |  |
| rno-miR-185-5p | | 8246.0 | 8202.3 |  |  |
| rno-miR-872-5p | | 8030.3 | 6895.2 |  |  |
| rno-miR-148b-3p | | 7583.1 | 6476.2 |  |  |
| rno-miR-182 | | 7395.2 | 7503.9 |  |  |
| rno-miR-28-3p | | 7255.6 | 6216.1 |  |  |
| rno-miR-872-3p | | 7193.3 | 7643.4 |  |  |
| rno-miR-328a-3p | | 6853.4 | 7425.5 |  |  |
| rno-miR-339-5p | | 6377.1 | 6108.1 |  |  |
| rno-miR-221-3p | | 5663.8 | 6616.1 |  |  |
| rno-miR-140-3p | | 4844.0 | 4807.1 |  |  |
| rno-miR-451-3p | | 4841.8 | 5371.8 |  |  |
| rno-miR-15b-3p | | 4809.0 | 4126.3 |  |  |
| rno-miR-101b-3p | | 4617.7 | 4025.8 |  |  |
| rno-miR-122-5p | | 4471.7 | 3250.0 |  |  |
| rno-miR-339-3p | | 4432.0 | 4492.1 |  |  |
| rno-miR-652-3p | | 4419.2 | 4765.1 |  |  |
| rno-miR-215 | | 4318.0 | 2689.0 |  |  |
| rno-miR-24-3p | | 4197.7 | 3974.4 |  |  |
| rno-miR-23a-3p | | 4168.9 | 4314.2 |  |  |
| rno-miR-130a-3p | | 4150.5 | 3615.9 |  |  |
| rno-miR-23b-3p | | 3931.7 | 3654.4 |  |  |
| rno-miR-181c-3p | | 3927.6 | 4075.2 |  |  |
| rno-miR-30a-3p | | 3595.5 | 3683.8 |  |  |
| rno-miR-148a-3p | | 3541.0 | 3722.3 |  |  |
| rno-miR-125a-5p | | 3178.5 | 3788.1 |  |  |
| rno-miR-342-3p | | 3110.9 | 2925.6 |  |  |
| rno-miR-140-5p | | 3098.7 | 2637.3 |  |  |
| rno-miR-194-5p | | 3024.4 | 2796.0 |  |  |
| rno-miR-363-3p | | 3003.1 | 2857.0 |  |  |
| rno-miR-3559-5p | | 2860.6 | 2510.7 |  |  |
| rno-miR-500-3p | | 2707.9 | 2853.5 |  |  |
| rno-miR-17-1-3p | | 2670.6 | 2934.7 |  |  |
| rno-miR-351-5p | | 2660.6 | 2635.2 |  |  |
| rno-miR-361-5p | | 2523.7 | 2639.3 |  |  |
| rno-miR-99b-5p | | 2460.2 | 2888.5 |  |  |
| rno-miR-32-5p | | 2372.6 | 2061.7 |  |  |
| rno-miR-1839-5p | | 2351.2 | 2270.8 |  |  |
| rno-miR-532-5p | | 2333.8 | 1974.8 |  |  |
| rno-miR-130b-3p | | 2287.5 | 2082.3 |  |  |
| rno-miR-181a-1-3p | | 2284.2 | 1819.3 |  |  |
| rno-miR-24-2-5p | | 2267.9 | 2290.4 |  |  |
| rno-miR-92b-3p | | 2163.4 | 1966.2 |  |  |
| rno-miR-3589 | | 2019.1 | 1923.1 |  |  |
| rno-miR-126a-3p | | 1895.7 | 1690.0 |  |  |
| rno-miR-146a-5p | | 1827.3 | 1986.2 |  |  |
| rno-miR-676 | | 1785.0 | 2053.1 |  |  |
| rno-miR-28-5p | | 1522.2 | 1384.4 |  |  |
| rno-miR-3473 | | 1482.1 | 1843.6 |  |  |
| rno-miR-532-3p | | 1481.3 | 1587.1 |  |  |
| rno-miR-21-3p | | 1392.7 | 1170.5 |  |  |
| rno-miR-93-3p | | 1267.5 | 1301.7 |  |  |
| rno-miR-449a-5p | | 1258.4 | 1048.6 |  |  |
| rno-miR-326-3p | | 1252.6 | 1169.1 |  |  |
| rno-miR-374-5p | | 1200.0 | 1113.5 |  |  |
| rno-miR-450a-5p | | 1056.2 | 750.2 |  |  |
| rno-miR-322-5p | | 1004.1 | 708.6 |  |  |
| rno-miR-30c-2-3p | | 980.6 | 1187.6 |  |  |
| rno-let-7i-3p | | 945.8 | 1047.6 |  |  |
| rno-miR-155-5p | | 912.2 | 592.4 |  |  |
| rno-miR-34c-5p | | 901.0 | 969.8 |  |  |
| rno-let-7e-5p | | 899.3 | 866.9 |  |  |
| rno-miR-138-5p | | 861.2 | 1089.7 |  |  |
| rno-miR-501-3p | | 845.6 | 770.2 |  |  |
| rno-miR-298-5p | | 832.8 | 1081.8 |  |  |
| rno-miR-1843a-5p | | 775.5 | 792.6 |  |  |
| rno-miR-365-3p | | 766.1 | 909.8 |  |  |
| rno-miR-1306-5p | | 753.0 | 746.3 |  |  |
| rno-miR-25-5p | | 736.0 | 864.5 |  |  |
| rno-miR-3590-3p | | 726.7 | 763.2 |  |  |
| rno-miR-191a-3p | | 723.9 | 690.8 |  |  |
| rno-miR-324-5p | | 717.5 | 833.4 |  |  |
| rno-miR-301b-3p | | 708.5 | 567.1 |  |  |
| rno-miR-361-3p | | 654.9 | 559.8 |  |  |
| rno-miR-488-3p | | 651.5 | 467.9 |  |  |
| rno-miR-674-3p | | 638.5 | 608.3 |  |  |
| rno-miR-30c-1-3p | | 637.4 | 550.9 |  |  |
| rno-miR-324-3p | | 628.6 | 666.3 |  |  |
| rno-miR-1-3p | | 617.1 | 448.3 |  |  |
| rno-miR-130b-5p | | 617.0 | 564.3 |  |  |
| rno-miR-145-5p | | 613.0 | 781.5 |  |  |
| rno-miR-199a-3p | | 609.6 | 927.1 |  |  |
| rno-miR-30d-3p | | 604.8 | 519.4 |  |  |
| rno-miR-26b-3p | | 596.1 | 608.1 |  |  |
| rno-miR-6329 | | 590.4 | 553.5 |  |  |
| rno-miR-125b-5p | | 583.3 | 708.1 |  |  |
| rno-miR-582-3p | | 580.4 | 684.3 |  |  |
| rno-miR-362-3p | | 579.4 | 431.8 |  |  |
| rno-miR-141-3p | | 572.0 | 569.2 |  |  |
| rno-miR-374-3p | | 567.7 | 468.2 |  |  |
| rno-miR-340-3p | | 554.0 | 596.9 |  |  |
| rno-miR-203a-3p | | 531.9 | 468.3 |  |  |
| rno-miR-3068-3p | | 531.3 | 511.2 |  |  |
| rno-miR-3553 | | 526.3 | 499.7 |  |  |
| rno-miR-31a-5p | | 495.4 | 501.6 |  |  |
| rno-miR-29c-3p | | 495.3 | 539.4 |  |  |
| rno-miR-378a-5p | | 488.6 | 499.4 |  |  |
| rno-miR-15a-5p | | 475.0 | 419.0 |  |  |
| rno-miR-147 | | 465.9 | 608.9 |  |  |
| rno-miR-362-5p | | 463.7 | 507.6 |  |  |
| rno-miR-92a-1-5p | | 455.2 | 280.0 |  |  |
| rno-miR-3559-3p | | 433.0 | 521.4 |  |  |
| rno-miR-99a-5p | | 409.2 | 443.1 |  |  |
| rno-miR-133a-3p | | 380.5 | 584.7 |  |  |
| rno-miR-497-5p | | 378.1 | 342.3 |  |  |
| rno-miR-183-5p | | 377.8 | 365.1 |  |  |
| rno-miR-1839-3p | | 372.5 | 322.2 |  |  |
| rno-miR-19a-5p | | 360.8 | 324.4 |  |  |
| rno-miR-29b-3p | | 346.0 | 274.6 |  |  |
| rno-miR-664-3p | | 339.3 | 305.7 |  |  |
| rno-miR-331-3p | | 330.9 | 309.8 |  |  |
| rno-miR-425-3p | | 317.5 | 331.5 |  |  |
| rno-miR-1843b-5p | | 316.0 | 402.5 |  |  |
| rno-miR-350 | | 312.1 | 328.6 |  |  |
| rno-miR-33-5p | | 290.6 | 212.8 |  |  |
| rno-miR-101a-5p | | 285.4 | 281.1 |  |  |
| rno-miR-27b-5p | | 281.3 | 249.8 |  |  |
| rno-miR-18a-3p | | 267.5 | 339.5 |  |  |
| rno-miR-188-5p | | 266.7 | 257.3 |  |  |
| rno-miR-375-3p | | 266.2 | 481.2 |  |  |
| rno-miR-16-3p | | 228.5 | 195.5 |  |  |
| rno-miR-125b-2-3p | | 223.3 | 238.9 |  |  |
| rno-miR-505-3p | | 220.3 | 218.9 |  |  |
| rno-miR-196b-5p | | 213.4 | 238.0 |  |  |
| rno-miR-342-5p | | 205.9 | 179.4 |  |  |
| rno-miR-100-5p | | 201.8 | 275.9 |  |  |
| rno-miR-7a-5p | | 197.3 | 218.7 |  |  |
| rno-miR-193a-3p | | 194.2 | 314.8 |  |  |
| rno-miR-345-3p | | 189.3 | 210.6 |  |  |
| rno-miR-222-3p | | 188.9 | 204.6 |  |  |
| rno-miR-20b-5p | | 179.7 | 170.8 |  |  |
| rno-miR-127-3p | | 172.9 | 168.3 |  |  |
| rno-miR-330-5p | | 168.4 | 211.8 |  |  |
| rno-miR-328b-3p | | 166.8 | 225.0 |  |  |
| rno-miR-139-5p | | 155.2 | 139.2 |  |  |
| rno-miR-34a-5p | | 154.1 | 129.1 |  |  |
| rno-miR-210-5p | | 153.1 | 137.5 |  |  |
| rno-miR-345-5p | | 151.3 | 122.6 |  |  |
| rno-let-7b-3p | | 145.1 | 113.9 |  |  |
| rno-miR-3577 | | 144.4 | 156.5 |  |  |
| rno-miR-338-3p | | 139.7 | 152.9 |  |  |
| rno-miR-148b-5p | | 133.3 | 111.5 |  |  |
| rno-miR-195-5p | | 131.4 | 102.1 |  |  |
| rno-miR-505-5p | | 130.3 | 135.4 |  |  |
| rno-miR-3558-5p | | 129.5 | 107.9 |  |  |
| rno-miR-145-3p | | 126.2 | 135.2 |  |  |
| rno-miR-1249 | | 123.2 | 163.4 |  |  |
| rno-miR-223-5p | | 118.3 | 139.9 |  |  |
| rno-miR-3068-5p | | 111.8 | 108.3 |  |  |
| rno-miR-674-5p | | 109.1 | 112.0 |  |  |
| rno-miR-6315 | | 108.8 | 81.8 |  |  |
| rno-miR-152-3p | | 106.9 | 201.6 |  |  |
| rno-miR-3074 | | 104.4 | 83.8 |  |  |
| rno-miR-7a-1-3p | | 103.6 | 93.5 |  |  |
| rno-miR-1956-5p | | 103.1 | 114.7 |  |  |
| rno-let-7a-1-3p | | 102.8 | 81.7 |  |  |
| rno-miR-9a-5p | | 101.5 | 91.1 |  |  |
| rno-let-7c-2-3p | | 98.3 | 75.7 |  |  |
| rno-miR-6324 | | 98.3 | 140.5 |  |  |
| rno-miR-1306-3p | | 98.2 | 87.4 |  |  |
| rno-miR-330-3p | | 97.2 | 101.6 |  |  |
| rno-miR-296-5p | | 94.9 | 121.9 |  |  |
| rno-let-7f-1-3p | | 92.6 | 66.5 |  |  |
| rno-miR-434-3p | | 92.4 | 140.9 |  |  |
| rno-miR-27a-5p | | 91.6 | 102.9 |  |  |
| rno-miR-671 | | 90.6 | 99.1 |  |  |
| rno-miR-22-5p | | 89.1 | 122.3 |  |  |
| rno-miR-542-3p | | 83.3 | 61.5 |  |  |
| rno-miR-128-1-5p | | 78.4 | 103.4 |  |  |
| rno-let-7g-3p | | 74.9 | 82.2 |  |  |
| rno-miR-702-3p | | 72.8 | 112.6 |  |  |
| rno-miR-30b-3p | | 71.5 | 58.0 |  |  |
| rno-miR-132-3p | | 68.6 | 88.5 |  |  |
| rno-miR-149-5p | | 68.1 | 94.3 |  |  |
| rno-miR-335 | | 65.4 | 65.1 |  |  |
| rno-miR-181a-2-3p | | 63.6 | 75.9 |  |  |
| rno-miR-219a-1-3p | | 60.2 | 52.6 |  |  |
| rno-miR-877 | | 59.3 | 45.9 |  |  |
| rno-miR-199a-5p | | 58.8 | 142.9 |  |  |
| rno-miR-204-5p | | 57.5 | 55.1 |  |  |
| rno-miR-1b | | 57.1 | 29.6 |  |  |
| rno-miR-32-3p | | 55.7 | 16.0 |  |  |
| rno-miR-3075 | | 55.7 | 41.1 |  |  |
| rno-miR-499-5p | | 52.6 | 27.8 |  |  |
| rno-miR-450b-5p | | 52.3 | 29.4 |  |  |
| rno-miR-187-3p | | 49.1 | 41.8 |  |  |
| rno-miR-542-5p | | 49.0 | 42.1 |  |  |
| rno-miR-205 | | 48.9 | 136.8 |  |  |
| rno-miR-33-3p | | 44.2 | 36.9 |  |  |
| rno-miR-503-5p | | 43.0 | 41.0 |  |  |
| rno-miR-3590-5p | | 41.8 | 67.6 |  |  |
| rno-miR-6216 | | 39.6 | 33.7 |  |  |
| rno-miR-582-5p | | 38.8 | 39.5 |  |  |
| rno-miR-652-5p | | 34.5 | 35.6 |  |  |
| rno-miR-672-5p | | 33.9 | 42.4 |  |  |
| rno-miR-6319 | | 33.5 | 33.7 |  |  |
| rno-miR-34b-5p | | 32.2 | 28.3 |  |  |
| rno-miR-221-5p | | 29.4 | 44.4 |  |  |
| rno-miR-96-5p | | 29.2 | 30.1 |  |  |
| rno-miR-5132-5p | | 29.0 | 16.9 |  |  |
| rno-miR-1843a-3p | | 27.8 | 25.1 |  |  |
| rno-miR-219a-5p | | 27.4 | 34.9 |  |  |
| rno-miR-200c-3p | | 26.1 | 25.0 |  |  |
| rno-miR-466c-5p | | 26.0 | 31.8 |  |  |
| rno-miR-429 | | 25.5 | 66.8 |  |  |
| rno-miR-455-5p | | 25.1 | 21.9 |  |  |
| rno-miR-17-2-3p | | 24.6 | 18.6 |  |  |
| rno-miR-6318 | | 24.4 | 38.6 |  |  |
| rno-miR-6328 | | 24.0 | 23.2 |  |  |
| rno-miR-133b-3p | | 23.0 | 72.3 |  |  |
| rno-miR-352 | | 22.0 | 34.3 |  |  |
| rno-miR-489-3p | | 20.7 | 18.5 |  |  |
| rno-miR-200a-3p | | 20.2 | 46.0 |  |  |
| rno-miR-219b | | 20.0 | 38.0 |  |  |
| rno-miR-1298 | | 19.9 | 23.2 |  |  |
| rno-miR-212-3p | | 19.9 | 20.3 |  |  |
| rno-miR-338-5p | | 19.8 | 28.7 |  |  |
| rno-miR-541-5p | | 19.4 | 17.8 |  |  |
| rno-miR-511-3p | | 19.1 | 33.1 |  |  |
| rno-miR-181b-1-3p | | 19.1 | 4.7 |  |  |
| rno-miR-191b | | 19.0 | 20.4 |  |  |
| rno-miR-653-5p | | 18.7 | 21.9 |  |  |
| rno-miR-500-5p | | 17.8 | 23.6 |  |  |
| rno-miR-20b-3p | | 17.8 | 15.9 |  |  |
| rno-miR-98-3p | | 17.7 | 12.1 |  |  |
| rno-miR-195-3p | | 17.2 | 10.2 |  |  |
| rno-miR-874-3p | | 16.3 | 22.4 |  |  |
| rno-miR-152-5p | | 15.4 | 23.3 |  |  |
| rno-miR-802-3p | | 15.0 | 9.0 |  |  |
| rno-miR-200b-3p | | 14.7 | 28.7 |  |  |
| rno-miR-99b-3p | | 14.7 | 10.0 |  |  |
| rno-miR-615 | | 13.7 | 7.4 |  |  |
| rno-miR-511-5p | | 13.4 | 13.5 |  |  |
| rno-miR-501-5p | | 13.3 | 18.5 |  |  |
| rno-miR-107-5p | | 13.2 | 6.8 |  |  |
| rno-miR-133a-5p | | 12.6 | 39.5 |  |  |
| rno-miR-34b-3p | | 12.4 | 14.8 |  |  |
| rno-miR-211-5p | | 12.4 | 54.8 |  |  |
| rno-miR-455-3p | | 12.2 | 2.5 |  |  |
| rno-miR-1224 | | 12.0 | 17.5 |  |  |
| rno-miR-24-1-5p | | 11.6 | 21.6 |  |  |
| rno-miR-503-3p | | 11.5 | 17.3 |  |  |
| rno-miR-203b-3p | | 11.5 | 16.2 |  |  |
| rno-miR-10a-3p | | 11.5 | 19.0 |  |  |
| rno-miR-150-3p | | 11.4 | 20.5 |  |  |
| rno-miR-298-3p | | 11.4 | 19.9 |  |  |
| rno-miR-410-3p | | 11.2 | 18.9 |  |  |
| rno-miR-411-5p | | 10.9 | 17.9 |  |  |
| rno-miR-421-5p | | 10.6 | 8.5 |  |  |
| rno-miR-218a-5p | | 10.5 | 7.2 |  |  |
| rno-miR-6321 | | 10.3 | 18.9 |  |  |
| rno-miR-322-3p | | 10.3 | 9.2 |  |  |
| rno-miR-19b-1-5p | | 9.8 | 8.6 |  |  |
| rno-miR-184 | | 9.8 | 12.7 |  |  |
| rno-miR-136-3p | | 9.6 | 19.7 |  |  |
| rno-miR-3593-5p | | 9.5 | 2.6 |  |  |
| rno-miR-125b-1-3p | | 9.5 | 13.7 |  |  |
| rno-let-7f-2-3p | | 9.4 | 15.3 |  |  |
| rno-miR-29a-5p | | 9.3 | 16.2 |  |  |
| rno-miR-3064-3p | | 9.0 | 22.7 |  |  |
| rno-miR-421-3p | | 8.9 | 9.2 |  |  |
| rno-miR-3084b-3p | | 8.8 | 9.0 |  |  |
| rno-miR-122-3p | | 8.3 | 2.1 |  |  |
| rno-miR-1949 | | 8.3 | 2.9 |  |  |
| rno-miR-3572 | | 8.0 | 3.8 |  |  |
| rno-miR-378b | | 8.0 | 9.0 |  |  |
| rno-miR-409b | | 7.9 | 12.1 |  |  |
| rno-miR-208a-5p | | 7.8 | 2.1 |  |  |
| rno-miR-3084a-3p | | 7.8 | 8.2 |  |  |
| rno-miR-10b-5p | | 7.8 | 3.4 |  |  |
| rno-miR-20a-3p | | 7.7 | 11.0 |  |  |
| rno-miR-141-5p | | 7.6 | 6.2 |  |  |
| rno-miR-186-3p | | 7.5 | 6.0 |  |  |
| rno-miR-802-5p | | 7.2 | 2.1 |  |  |
| rno-miR-3064-5p | | 7.0 | 4.9 |  |  |
| rno-miR-183-3p | | 7.0 | 6.2 |  |  |
| rno-miR-490-3p | | 6.8 | 19.9 |  |  |
| rno-miR-136-5p | | 6.7 | 13.0 |  |  |
| rno-miR-181d-3p | | 6.7 | 1.5 |  |  |
| rno-miR-99a-3p | | 6.6 | 6.7 |  |  |
| rno-miR-3084d | | 6.5 | 7.7 |  |  |
| rno-miR-125a-3p | | 6.5 | 10.2 |  |  |
| rno-miR-664-2-5p | | 6.1 | 4.8 |  |  |
| rno-miR-653-3p | | 6.0 | 6.3 |  |  |
| rno-miR-3585-5p | | 5.8 | 0.5 |  |  |
| rno-miR-34c-3p | | 5.8 | 9.4 |  |  |
| rno-miR-301a-5p | | 5.8 | 20.0 |  |  |
| rno-miR-190a-5p | | 5.6 | 4.9 |  |  |
| rno-miR-214-3p | | 5.6 | 15.8 |  |  |
| rno-miR-466b-5p | | 5.5 | 3.0 |  |  |
| rno-miR-224-5p | | 5.3 | 7.8 |  |  |
| rno-miR-190b-5p | | 5.2 | 3.9 |  |  |
| rno-miR-29b-5p | | 5.1 | 4.4 |  |  |
| rno-miR-101b-5p | | 5.1 | 3.1 |  |  |
| rno-miR-188-3p | | 5.1 | 5.2 |  |  |
| rno-miR-1843b-3p | | 5.0 | 2.5 |  |  |
| rno-miR-193b-3p | | 4.9 | 23.0 |  |  |
| rno-miR-9a-3p | | 4.9 | 3.9 |  |  |
| rno-miR-384-5p | | 4.8 | 0.0 |  |  |
| rno-miR-3084b-5p | | 4.8 | 2.9 |  |  |
| rno-miR-3583-5p | | 4.8 | 2.1 |  |  |
| rno-let-7e-3p | | 4.8 | 7.2 |  |  |
| rno-miR-192-3p | | 4.8 | 8.9 |  |  |
| rno-miR-3065-3p | | 4.4 | 6.9 |  |  |
| rno-miR-3583-3p | | 4.4 | 1.9 |  |  |
| rno-miR-132-5p | | 4.3 | 3.7 |  |  |
| rno-miR-293-5p | | 4.1 | 0.0 |  |  |
| rno-miR-383-5p | | 4.1 | 3.6 |  |  |
| rno-miR-5132-3p | | 4.1 | 4.4 |  |  |
| rno-miR-103-1-5p | | 3.9 | 5.6 |  |  |
| rno-miR-3547 | | 3.9 | 1.0 |  |  |
| rno-miR-29c-5p | | 3.8 | 7.1 |  |  |
| rno-miR-1247-5p | | 3.7 | 8.6 |  |  |
| rno-miR-3542 | | 3.7 | 6.5 |  |  |
| rno-miR-504 | | 3.7 | 5.6 |  |  |
| rno-miR-383-3p | | 3.4 | 0.0 |  |  |
| rno-miR-212-5p | | 3.3 | 4.6 |  |  |
| rno-miR-487b-3p | | 3.3 | 3.2 |  |  |
| rno-miR-341 | | 3.0 | 3.3 |  |  |
| rno-miR-879-5p | | 3.0 | 2.8 |  |  |
| rno-miR-193a-5p | | 2.9 | 4.4 |  |  |
| rno-miR-873-5p | | 2.9 | 2.4 |  |  |
| rno-miR-3593-3p | | 2.8 | 6.0 |  |  |
| rno-miR-434-5p | | 2.6 | 9.7 |  |  |
| rno-miR-153-3p | | 2.6 | 1.7 |  |  |
| rno-miR-31a-3p | | 2.6 | 5.6 |  |  |
| rno-miR-127-5p | | 2.6 | 2.0 |  |  |
| rno-miR-382-5p | | 2.5 | 1.8 |  |  |
| rno-miR-466b-2-3p | | 2.4 | 2.4 |  |  |
| rno-miR-124-5p | | 2.4 | 1.8 |  |  |
| rno-miR-381-3p | | 2.4 | 0.0 |  |  |
| rno-miR-351-3p | | 2.4 | 1.5 |  |  |
| rno-miR-23a-5p | | 2.3 | 3.5 |  |  |
| rno-miR-139-3p | | 2.3 | 4.4 |  |  |
| rno-miR-134-5p | | 2.2 | 1.5 |  |  |
| rno-miR-296-3p | | 2.1 | 2.5 |  |  |
| rno-miR-489-5p | | 2.1 | 0.0 |  |  |
| rno-miR-146a-3p | | 2.1 | 0.9 |  |  |
| rno-miR-874-5p | | 2.1 | 3.4 |  |  |
| rno-miR-708-3p | | 2.1 | 6.7 |  |  |
| rno-miR-124-3p | | 2.0 | 0.0 |  |  |
| rno-miR-449c-5p | | 2.0 | 4.4 |  |  |
| rno-miR-7578 | | 2.0 | 4.7 |  |  |
| rno-miR-300-3p | | 2.0 | 12.3 |  |  |
| rno-miR-466b-3p | | 1.9 | 5.4 |  |  |
| rno-miR-547-5p | | 1.9 | 0.0 |  |  |
| rno-miR-138-2-3p | | 1.9 | 1.9 |  |  |
| rno-miR-222-5p | | 1.9 | 0.0 |  |  |
| rno-miR-146b-3p | | 1.9 | 0.5 |  |  |
| rno-miR-3120 | | 1.9 | 6.2 |  |  |
| rno-miR-331-5p | | 1.9 | 0.0 |  |  |
| rno-miR-483-3p | | 1.8 | 3.1 |  |  |
| rno-miR-129-5p | | 1.8 | 0.0 |  |  |
| rno-miR-196b-3p | | 1.8 | 1.7 |  |  |
| rno-miR-672-3p | | 1.8 | 1.0 |  |  |
| rno-miR-6334 | | 1.7 | 1.1 |  |  |
| rno-miR-194-3p | | 1.6 | 8.6 |  |  |
| rno-miR-760-3p | | 1.6 | 1.8 |  |  |
| rno-miR-181b-2-3p | | 1.5 | 0.0 |  |  |
| rno-miR-154-5p | | 1.4 | 0.0 |  |  |
| rno-miR-26a-3p | | 1.4 | 5.0 |  |  |
| rno-miR-294 | | 1.3 | 0.0 |  |  |
| rno-miR-337-3p | | 1.3 | 0.0 |  |  |
| rno-miR-344b-1-3p | | 1.3 | 0.0 |  |  |
| rno-miR-452-5p | | 1.3 | 0.0 |  |  |
| rno-miR-200a-5p | | 1.2 | 0.0 |  |  |
| rno-miR-224-3p | | 1.2 | 1.8 |  |  |
| rno-miR-295-3p | | 1.2 | 0.0 |  |  |
| rno-miR-301b-5p | | 1.2 | 3.2 |  |  |
| rno-miR-547-3p | | 1.2 | 0.0 |  |  |
| rno-miR-592 | | 1.2 | 0.0 |  |  |
| rno-miR-200b-5p | | 1.2 | 1.4 |  |  |
| rno-miR-299a-3p | | 1.2 | 0.0 |  |  |
| rno-miR-466b-4-3p | | 1.2 | 4.3 |  |  |
| rno-miR-196a-5p | | 1.2 | 1.1 |  |  |
| rno-miR-3102 | | 1.2 | 0.0 |  |  |
| rno-miR-433-3p | | 1.2 | 0.0 |  |  |
| rno-miR-6317 | | 1.2 | 0.0 |  |  |
| rno-miR-196c-5p | | 1.1 | 0.0 |  |  |
| rno-miR-344b-5p | | 1.1 | 0.5 |  |  |
| rno-miR-379-3p | | 1.1 | 0.0 |  |  |
| rno-miR-540-3p | | 1.1 | 0.0 |  |  |
| rno-miR-764-3p | | 1.1 | 0.0 |  |  |
| rno-miR-135b-5p | | 0.9 | 0.0 |  |  |
| rno-miR-218a-1-3p | | 0.9 | 1.0 |  |  |
| rno-miR-3566 | | 0.9 | 0.0 |  |  |
| rno-miR-370-3p | | 0.9 | 1.8 |  |  |
| rno-miR-92b-5p | | 0.9 | 0.0 |  |  |
| rno-miR-143-5p | | 0.7 | 5.4 |  |  |
| rno-miR-155-3p | | 0.7 | 0.0 |  |  |
| rno-miR-187-5p | | 0.7 | 0.0 |  |  |
| rno-miR-201-5p | | 0.7 | 0.0 |  |  |
| rno-miR-3562 | | 0.7 | 0.0 |  |  |
| rno-miR-3584-5p | | 0.7 | 0.0 |  |  |
| rno-miR-431 | | 0.7 | 3.0 |  |  |
| rno-miR-450b-3p | | 0.7 | 3.1 |  |  |
| rno-miR-6314 | | 0.7 | 0.0 |  |  |
| rno-miR-873-3p | | 0.7 | 1.8 |  |  |
| rno-miR-1247-3p | | 0.7 | 0.5 |  |  |
| rno-miR-129-2-3p | | 0.7 | 0.0 |  |  |
| rno-miR-130a-5p | | 0.7 | 1.6 |  |  |
| rno-miR-134-3p | | 0.7 | 0.9 |  |  |
| rno-miR-135a-5p | | 0.7 | 0.0 |  |  |
| rno-miR-15a-3p | | 0.7 | 1.0 |  |  |
| rno-miR-203a-5p | | 0.7 | 0.5 |  |  |
| rno-miR-216a-5p | | 0.7 | 6.5 |  |  |
| rno-miR-3550 | | 0.7 | 0.0 |  |  |
| rno-miR-3569 | | 0.7 | 1.0 |  |  |
| rno-miR-3594-3p | | 0.7 | 0.0 |  |  |
| rno-miR-363-5p | | 0.7 | 0.0 |  |  |
| rno-miR-369-5p | | 0.7 | 0.5 |  |  |
| rno-miR-380-3p | | 0.7 | 0.0 |  |  |
| rno-miR-448-3p | | 0.7 | 2.1 |  |  |
| rno-miR-540-5p | | 0.7 | 3.3 |  |  |
| rno-miR-6215 | | 0.7 | 0.0 |  |  |
| rno-miR-668 | | 0.7 | 0.5 |  |  |
| rno-miR-743b-3p | | 0.7 | 1.6 |  |  |
| rno-miR-875 | | 0.7 | 0.0 |  |  |
| rno-miR-935 | | 0.7 | 0.0 |  |  |
| rno-let-7a-2-3p | | 0.0 | 1.0 |  |  |
| rno-miR-128-2-5p | | 0.0 | 1.1 |  |  |
| rno-miR-193b-5p | | 0.0 | 1.8 |  |  |
| rno-miR-206-3p | | 0.0 | 74.4 |  |  |
| rno-miR-207 | | 0.0 | 1.0 |  |  |
| rno-miR-208a-3p | | 0.0 | 2.9 |  |  |
| rno-miR-217-3p | | 0.0 | 1.8 |  |  |
| rno-miR-217-5p | | 0.0 | 1.2 |  |  |
| rno-miR-325-3p | | 0.0 | 2.2 |  |  |
| rno-miR-329-3p | | 0.0 | 1.2 |  |  |
| rno-miR-329-5p | | 0.0 | 4.1 |  |  |
| rno-miR-336-5p | | 0.0 | 1.5 |  |  |
| rno-miR-346 | | 0.0 | 3.1 |  |  |
| rno-miR-3558-3p | | 0.0 | 0.5 |  |  |
| rno-miR-3588 | | 0.0 | 0.5 |  |  |
| rno-miR-369-3p | | 0.0 | 2.7 |  |  |
| rno-miR-488-5p | | 0.0 | 0.5 |  |  |
| rno-miR-493-5p | | 0.0 | 0.9 |  |  |
| rno-miR-494-3p | | 0.0 | 0.9 |  |  |
| rno-miR-499-3p | | 0.0 | 2.7 |  |  |
| rno-miR-551b-3p | | 0.0 | 0.5 |  |  |
| rno-miR-666-3p | | 0.0 | 0.5 |  |  |
| rno-miR-702-5p | | 0.0 | 2.4 |  |  |
| rno-miR-743a-5p | | 0.0 | 1.5 |  |  |
| rno-miR-743b-5p | | 0.0 | 0.5 |  |  |
| rno-miR-7a-2-3p | | 0.0 | 1.8 |  |  |
| rno-miR-881-3p | | 0.0 | 0.5 |  |  |
|  | | **Control** | **AMPH hyper** |  |  |
| **Total count:** | | 280230352.5 | 301933649.9 |  |  |
